# Frontiers of optic nerve regeneration research: an analysis of the top 100 most influential articles in the field from 2005 to 2025

**DOI:** 10.3389/fnins.2025.1634999

**Published:** 2025-09-25

**Authors:** Saijilafu, Peng Chen, Lingchen Ye, Yuxi Shen, Qi Wang, Xuanwen Chen, Chimedragchaa Chimedtseren, Junqian Zhang, Linjun Fang, Renjie Xu

**Affiliations:** ^1^Hangzhou Lin’an Traditional Chinese Medicine Hospital, Affiliated Hospital, Hangzhou City University, Hangzhou, Zhejiang, China; ^2^Key Laboratory of Novel Targets and Drug Study for Neural Repair of Zhejiang Province, School of Medicine, Hangzhou City University, Hangzhou, China; ^3^Department of Orthopedics, Suzhou Municipal Hospital, The Affiliated Suzhou Hospital of Nanjing Medical University, Suzhou, China; ^4^Institute of Traditional Medicine and Technology of Mongolia, Ulaanbaatar, Mongolia

**Keywords:** optic nerve regeneration, bibliometric analysis, web of science core collection, VOS viewer, CiteSpace

## Abstract

**Objectives:**

In this study, we evaluated the key features of the 100 most-cited publications on optic nerve regeneration from 2005 to 2025 employing bibliometric and visual analysis.

**Methods:**

The data for this study were obtained from a comprehensive search across multiple databases, including the Web of Science, Scopus, and Dimensions. We identified the top 100 most-cited articles published in each database from 2005 to 2025, merged and deduplicated the results, and selected the 100 most-cited papers on optic nerve regeneration. After extracting key details such as titles, authors, keywords, publication information, and institutional affiliations, a bibliometric analysis was conducted.

**Results:**

The top 100 most cited papers on optic nerve regeneration published between 2005 and 2025, accumulating 34,636 total citations with a median of 346 citations per paper. Prof. Zhigang He emerged as the most prolific author with 19 publications. The United States contributed 59 papers, while Harvard University led institutions with 30 publications. Key research themes included optic nerve regeneration, CNTF, gene therapy, and retinal ganglion cells.

**Conclusion:**

Our analysis of top-cited optic nerve regeneration research reveals sustained United States leadership in output and innovation. Early work focused on neuronal signaling pathways (PTEN/mTOR, KLF family), while current studies explore novel targets and biomaterials. Global collaboration among the United States, China, and European nations has accelerated progress. Key challenges remain in achieving functional long-distance regeneration. Future direction should prioritize the development of multi-target therapeutic methods, precise drug delivery, and the control of inflammation to improve nerve regeneration efficiency.

## 1 Introduction

The optic nerve, comprising the axons of retinal ganglion cells (RGCs), is the only pathway through which visual signals travel from the retina to the brain, and whose functional integrity is essential for maintaining visual perception (Laha et al., 2017). The optic nerve is a crucial component of the central nervous system (CNS) and, as such, shares the limited regenerative capacity characteristic of the mature CNS of most mammals. Additionally, its axons are prone to irreversible degenerative changes after injury, and their regenerative capacity is significantly lower than that of axons in the peripheral nervous system (PNS) (Benowitz et al., 2017). Traumatic optic neuropathy, genetic disorders, and diseases such as glaucoma, can result in damage to the optic nerve (Chen et al., 2022). Such damage can not only severely impair visual function, but can also directly lead to the apoptosis of RGCs, and, eventually, irreversible blindness.

Optic nerve regeneration is an important prerequisite for the recovery of visual function. Despite this, how to promote this process remains a major challenge in the field of neuroscience. Current research efforts are focused on decoding the intrinsic regulatory mechanisms of RGCs (Moore et al., 2009; Park et al., 2008), the modulation of the neural microenvironment (Yin et al., 2006; Yin et al., 2009), guiding axon growth, and the restoration of visual function. A range of methods are employed at present for inducing optic nerve regeneration, including multi-gene therapy (Kurimoto et al., 2010), immune system modulation (Baldwin et al., 2015), neurotrophic factor therapy (Bei et al., 2016; Jacobi et al., 2022; Müller et al., 2007), cell therapy (Mead et al., 2015; Mead et al., 2017), and bioactive material-based strategies (Pan et al., 2024).

Bibliometric analysis, supported by visualization methods, can help researchers understand the development and research hotspots within academic fields. Bibliometric methods, mainly involving literature quantity, collaboration, influence, and keyword analysis, are increasingly used in medicine. However, bibliometric studies relating to optic nerve research are relatively scarce, and there is a need to clarify the current situation, hot spots, and trends in this field. The aim of this study was to comprehensively analyze the 100 most frequently cited papers on optic nerve regeneration published between 2005 and 2025,and construct a relevant multi-dimensional knowledge map comprising an international cooperation network, a core author cluster, data on high-impact journal distribution, and a keyword co-occurrence network.

## 2 Materials and methods

### 2.1 Search strategies and data extraction

**FIGURE 1:**
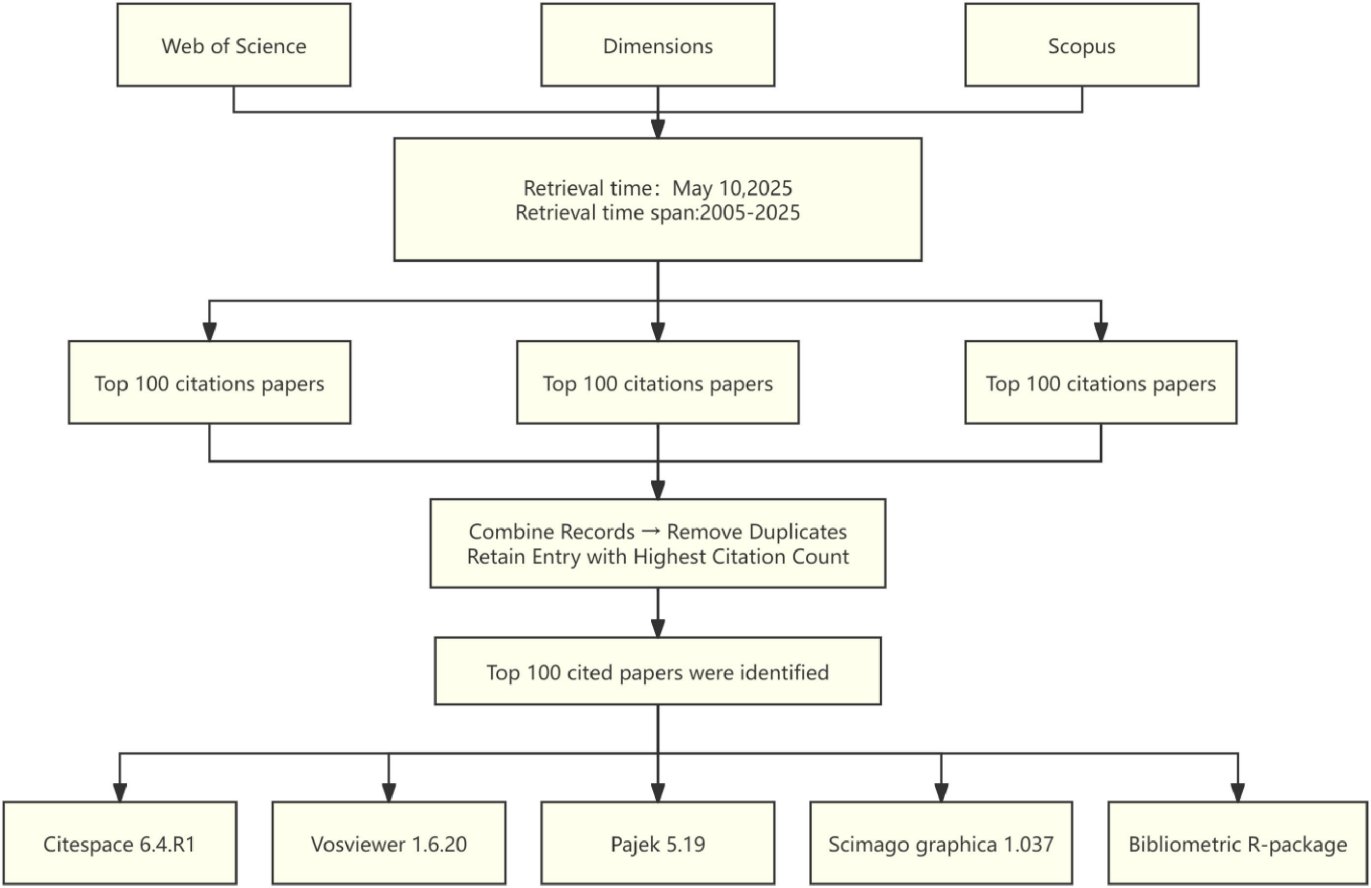
Document screening flow chart.

The data for this study were sourced from three authoritative databases: the Web of Science Core Collection (WoSCC), Scopus, and Dimensions. WoSCC is distinguished by its stringent journal selection criteria, while Scopus provides the most extensive disciplinary coverage. Additionally, Dimensions integrate various types of research data. The combined utilization of these databases ensures comprehensive and representative literature retrieval process. Articles and reviews published between 2005 and 2025, were retrieved using the following search terms: (optic nerve regeneration*) or (Optic nerve repair*) or (Optic nerve recovery*) or (Optic nerve regrowth*) or (Axonal regeneration of retinal ganglion cells); the language was limited to English. The top 100 most-cited articles were retrieved separately from each database, with data downloaded on 10 May 2025, to prevent potential bias from subsequent database updates. After merging and removing duplicate records, the final list of top 100 most-cited articles was determined based on citation frequency ranking. In addition, two researchers independently screened the titles, abstracts, and document types. In case of disagreement, the full text of the manuscript was reviewed, and consensus was reached through discussion. The final dataset was exported in the “Complete Record and Cited References” format for subsequent analysis, including title, authors, keywords, journal, publication year, country, and institutional affiliation (Figure 1).

### 2.2 Data analysis and visualization

In this study, several bibliometric tools were used to systematically analyze the data. Initially, descriptive statistical analysis of basic data and visual chart generation were performed using Microsoft Excel 2019. Then, a keyword co-occurrence network and an author cooperation network were constructed using VOS viewer, a document measurement tool developed by van Eck and Waltman (2010). These visualizations intuitively illustrate the clustering of research hotspots and academic cooperation relationships, respectively. Furthermore, in conjunction with the network analysis capabilities of Pajek (Batagelj and Mrvar, 2002), a domain focus map was generated to reveal the core direction of current research. Meanwhile, a map of global academic influence was drawn using Scimago Graphica (Hassan-Montero et al., 2022), showing the citation frequency of the literature in different countries/regions in the form of geographical distribution. Additionally, CiteSpace (Chen, 2004) software was employed for multi-dimensional dynamic analysis, enabling the identification of academic communities based on author cooperation networks, the detection of keywords with high burst intensity to track the research frontier, and the visualization of the evolution of research hotspots through a timeline graph. In the network graph generated by CiteSpace, institutions, authors, and keywords are represented as nodes. The thickness of the lines between nodes reflects the cooperation intensity and co-occurrence frequency. Clustering groups, distinguished by different colors, represent subdivisions within the research topic. In addition, Bibliometrix, an R language toolkit, was used for bibliometric index analysis, enabling the rapid identification of the field’s founding literature, high-impact researchers, and emerging research directions. The tool also supports the generation of Sankey diagrams, where the arrow or line width represents the magnitude of knowledge flow paths, such as cross-disciplinary strength or inheritance of research topics, thereby providing visual support for data-driven interpretation of academic trends.

## 3 Results

### 3.1 Analysis of publications and citations

**FIGURE 2:**
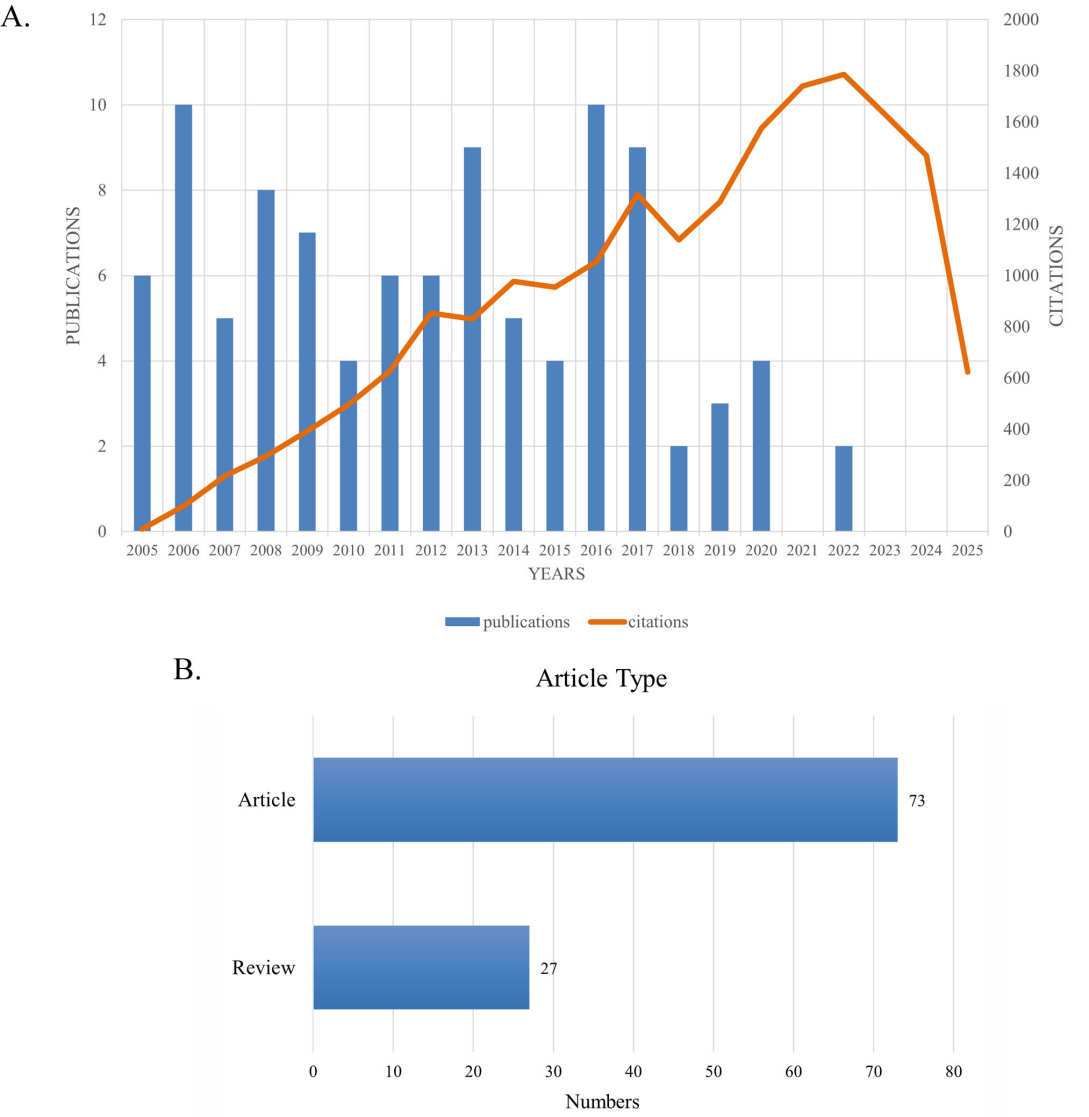
**(A)** Number and citations of top-cited publications from 2005 to 2025. **(B)** Article types and quantities.

Figure 2A presents the number of published articles and citation counts from 2005 to 2025. Figure 2B shows that among these top 100 most cited articles, research articles account for approximately three quarters and reviews for about one quarter. The details of the top 100 most-cited articles on optic nerve regeneration from 2005 to 2025 are presented in Table 1. The top 100 articles accumulated between 103 and 1,545 citations, with a median of 169.5 and an average of 346.4 citations per article. The most cited article, “Promoting Axon Regeneration in the Adult CNS by Modulation of the PTEN/mTOR Pathway” (Park et al., 2008), was published in SCIENCE in 2008 and has been cited 1,545 times. The second most cited paper, “Nano neuro knitting: Peptide nanofiber scaffold for brain repair and axon regeneration with functional return of vision” (Ellis-Behnke et al., 2006), appeared in the journal PROCEEDINGS OF THE NATIONAL ACADEMY OF SCIENCES OF THE UNITED STATES OF AMERICA in 2009 and has garnered 790 citations. The third most-cited paper, “Sustained axon regeneration induced by co-deletion of PTEN and SOCS3” (Sun et al., 2011), was published in NATURE in 2011 and accumulated 690 citations. While earlier publications have higher total citation counts, when ranked according to the average number of citations per year, some later publications were found to have a greater impact. For example, “Reprogramming to recover youthful epigenetic information and restore vision” (Tran et al., 2019), published in NATURE in 2020, ranked Fifth in total citations and First in average citations per year (124.0).

**TABLE 1:** The 100 most-cited articles in the field of optic nerve regeneration (2005–2025).

Number	First authors	Publication year	Title	Source title	Total citations	Average citations per year
1	Park, KK	2008	Promoting axon regeneration in the adult CNS by modulation of the PTEN/mTOR pathway (Park et al., 2008)	SCIENCE	1,545	90.9
2	Ellis-Behnke, RG	2006	Nano neuro knitting: peptide nanofiber scaffold for brain repair and axon regeneration with functional return of vision (Ellis-Behnke et al., 2006)	PROCEEDINGS OF THE NATIONAL ACADEMY OF SCIENCES OF THE UNITED STATES OF AMERICA	790	41.6
3	Sun, F	2011	Sustained axon regeneration induced by co-deletion of PTEN and SOCS3 (Sun et al., 2011)	NATURE	690	49.3
4	Moore, DL	2009	KLF family members regulate intrinsic axon regeneration ability (Moore et al., 2009)	SCIENCE	678	42.4
5	Lu, YC	2020	Reprogramming to recover youthful epigenetic information and restore vision (Lu et al., 2020)	NATURE	622	124.4
6	Tran, NM	2019	Single-cell profiles of retinal ganglion cells differing in resilience to injury reveal neuroprotective genes (Tran et al., 2019)	NEURON	540	90.0
7	Yin, YQ	2006	Oncomodulin is a macrophage-derived signal for axon regeneration in retinal ganglion cells (Yin et al., 2006)	NATURE NEUROSCIENCE	501	26.4
8	He, ZG	2016	Intrinsic control of axon regeneration (He and Jin, 2016)	NEURON	494	54.9
9	Smith, PD	2009	SOCS3 deletion promotes optic nerve regeneration *in vivo* (Smith et al., 2009)	NEURON	484	30.3
10	Duan, X	2015	Subtype-specific regeneration of retinal ganglion cells following axotomy: effects of osteopontin and mTOR signaling (Duan et al., 2015)	NEURON	466	46.6
11	Dickendesher, TL	2012	NgR1 and NgR3 are receptors for chondroitin sulfate proteoglycans (Dickendesher et al., 2012)	NATURE NEUROSCIENCE	430	33.1
12	Liu, K	2011	Neuronal Intrinsic mechanisms of axon regeneration (Liu et al., 2011)	ANNUAL REVIEW OF NEUROSCIENCE, VOL 34	427	30.5
13	Fausett, BV	2006	A role for α1 tubulin-expressing Muller glia in regeneration of the injured zebrafish retina (Fausett and Goldman, 2006)	JOURNAL OF NEUROSCIENCE	402	21.2
14	Mead, B	2017	Bone marrow-derived mesenchymal stem cells-derived exosomes promote survival of retinal ganglion cells through miRNA-dependent mechanisms (Mead and Tomarev, 2017)	STEM CELLS TRANSLATIONAL MEDICINE	395	49.4
15	Koprivica, V	2005	EGFR activation mediates inhibition of axon regeneration by myelin and chondroitin sulfate proteoglycans (Koprivica et al., 2005)	SCIENCE	382	19.1
16	de Lima, S	2012	Full-length axon regeneration in the adult mouse optic nerve and partial recovery of simple visual behaviors (de Lima et al., 2012)	PROCEEDINGS OF THE NATIONAL ACADEMY OF SCIENCES OF THE UNITED STATES OF AMERICA	331	25.5
17	Schwab, ME	2014	Nogo limits neural plasticity and recovery from injury (Schwab and Strittmatter, 2014)	CURRENT OPINION IN NEUROBIOLOGY	329	29.9
18	Chandran, V	2016	A systems-level analysis of the peripheral nerve intrinsic axonal growth program (Chandran et al., 2016)	NEURON	305	33.9
19	Watkins, TA	2013	DLK initiates a transcriptional program that couples apoptotic and regenerative responses to axonal injury (Watkins et al., 2013)	PROCEEDINGS OF THE NATIONAL ACADEMY OF SCIENCES OF THE UNITED STATES OF AMERICA	305	25.4
20	Fimbel, SM	2007	Regeneration of inner retinal neurons after intravitreal injection of ouabain in zebrafish (Fimbel et al., 2007)	JOURNAL OF NEUROSCIENCE	292	16.2
21	Chang, EE	2012	Glaucoma 2.0: neuroprotection, neuroregeneration, neuroenhancement (Chang and Goldberg, 2012)	OPHTHALMOLOGY	291	22.4
22	Kurimoto, T	2010	Long-distance axon regeneration in the mature optic nerve: contributions of oncomodulin, cAMP, and pten gene deletion (Kurimoto et al., 2010)	JOURNAL OF NEUROSCIENCE	268	17.9
23	Leaver, SG	2006	AAV-mediated expression of CNTF promotes long-term survival and regeneration of adult rat retinal ganglion cells (Leaver et al., 2006)	GENE THERAPY	264	13.9
24	Lim, JHA	2016	Neural activity promotes long-distance, target-specific regeneration of adult retinal axons (Lim et al., 2016)	NATURE NEUROSCIENCE	260	28.9
25	Abe, N	2008	Nerve injury signaling (Abe and Cavalli, 2008)	CURRENT OPINION IN NEUROBIOLOGY	257	15.1
26	Fujita, Y	2014	Axon growth inhibition by RhoA/ROCK in the central nervous system (Fujita and Yamashita, 2014)	FRONTIERS IN NEUROSCIENCE	245	22.3
27	Leibinger, M	2009	Neuroprotective and axon growth-promoting effects following inflammatory stimulation on mature retinal ganglion cells in mice depend on ciliary neurotrophic factor and leukemia inhibitory factor (Leibinger et al., 2009)	JOURNAL OF NEUROSCIENCE	235	14.7
28	Müller, A	2007	Astrocyte-derived CNTF switches mature RGCs to a regenerative state following inflammatory stimulation (Müller et al., 2007)	BRAIN	233	12.9
29	Bei, FF	2016	Restoration of visual function by enhancing conduction in regenerated axons (Bei et al., 2016)	CELL	232	25.8
30	Belin, S	2015	Injury-induced decline of intrinsic regenerative ability revealed by quantitative proteomics (Belin et al., 2015)	NEURON	231	23.1
31	Sengottuvel, V	2011	Taxol facilitates axon regeneration in the mature CNS (Sengottuvel et al., 2011)	JOURNAL OF NEUROSCIENCE	231	16.5
32	Benowitz, LI	2011	Inflammation and axon regeneration (Benowitz and Popovich, 2011)	CURRENT OPINION IN NEUROLOGY	226	16.1
33	Lingor, P	2007	Inhibition of Rho kinase (ROCK) increases neurite outgrowth on chondroitin sulfate proteoglycan *in vitro* and axonal regeneration in the adult optic nerve *in vivo* (Lingor et al., 2007)	JOURNAL OF NEUROCHEMISTRY	221	12.3
34	Lingor, P	2008	ROCK inhibition and CNTF interact on intrinsic signaling pathways and differentially regulate survival and regeneration in retinal ganglion cells (Lingor et al., 2008)	BRAIN	218	12.8
35	Lambiase, A	2009	Experimental and clinical evidence of neuroprotection by nerve growth factor eye drops: Implications for glaucoma (Lambiase et al., 2009)	PROCEEDINGS OF THE NATIONAL ACADEMY OF SCIENCES OF THE UNITED STATES OF AMERICA	209	13.1
36	Benowitz, LI	2017	Reaching the brain: advances in optic nerve regeneration (Benowitz et al., 2017)	EXPERIMENTAL NEUROLOGY	200	25.0
37	Sun, F	2010	Neuronal intrinsic barriers for axon regeneration in the adult CNS (Sun and He, 2010)	CURRENT OPINION IN NEUROBIOLOGY	197	13.1
38	Mead, B	2013	Intravitreally transplanted dental pulp stem cells promote neuroprotection and axon regeneration of retinal ganglion cells after optic nerve injury (Mead et al., 2013)	INVESTIGATIVE OPHTHALMOLOGY & VISUAL SCIENCE	194	16.2
39	Zhou, FQ	2006	Intracellular control of developmental and regenerative axon growth (Zhou and Snider, 2006)	PHILOSOPHICAL TRANSACTIONS OF THE ROYAL SOCIETY B-BIOLOGICAL SCIENCES	193	10.2
40	Mead, B	2015	Stem cell treatment of degenerative eye disease (Mead et al., 2015)	STEM CELL RESEARCH	193	19.3
41	Sas, AR	2020	A new neutrophil subset promotes CNS neuron survival and axon regeneration (Sas et al., 2020)	NATURE IMMUNOLOGY	192	38.4
42	Yin, YQ	2009	Oncomodulin links inflammation to optic nerve regeneration (Yin et al., 2009)	PROCEEDINGS OF THE NATIONAL ACADEMY OF SCIENCES OF THE UNITED STATES OF AMERICA	187	11.7
43	Wareham, LK	2022	Solving neurodegeneration: common mechanisms and strategies for new treatments (Wareham et al., 2022)	MOLECULAR NEURODEGENERATION	187	62.3
44	Kurimoto, T	2013	Neutrophils express oncomodulin and promote optic nerve regeneration (Kurimoto et al., 2013)	JOURNAL OF NEUROSCIENCE	185	15.4
45	Bertrand, J	2005	Application of Rho antagonist to neuronal cell bodies promotes neurite growth in compartmented cultures and regeneration of retinal ganglion cell axons in the optic nerve of adult rats (Bertrand et al., 2005)	JOURNAL OF NEUROSCIENCE	181	9.1
46	Kretz, A	2005	Erythropoietin promotes regeneration of adult CNS neurons via Jak2/Stat3 and PI3K/Akt pathway activation (Kretz et al., 2005)	MOLECULAR AND CELLULAR NEUROSCIENCE	181	9.1
47	Norsworthy, MW	2017	Sox11 expression promotes regeneration of some retinal ganglion cell types but kills others (Norsworthy et al., 2017)	NEURON	181	22.6
48	Koch, JC	2014	ROCK2 is a major regulator of axonal degeneration, neuronal death and axonal regeneration in the CNS (Koch et al., 2014)	CELL DEATH & DISEASE	176	16.0
49	Veldman, MB	2007	Gene expression analysis of zebrafish retinal ganglion nerve regeneration identifies KLF6a and KLF7a regulators of axon regeneration cells during optic as important (Veldman et al., 2007)	DEVELOPMENTAL BIOLOGY	175	9.7
50	Lamba, D	2008	Neural regeneration and cell replacement: a view from the eye (Lamba et al., 2008)	CELL STEM CELL	171	10.1
51	Hilla, AM	2017	Microglia are irrelevant for neuronal degeneration and axon regeneration after acute injury (Hilla et al., 2017)	JOURNAL OF NEUROSCIENCE	168	21.0
52	Chidlow, G	2011	The optic nerve head is the site of axonal transport disruption, axonal cytoskeleton damage and putative axonal regeneration failure in a rat model of glaucoma (Chidlow et al., 2011)	ACTA NEUROPATHOLOGICA	161	11.5
53	Kimura, A	2016	Neuroprotection, growth factors and BDNF-TrkB signaling in retinal degeneration (Kimura et al., 2016)	INTERNATIONAL JOURNAL OF MOLECULAR SCIENCES	160	17.8
54	Singhal, S	2012	Human muller glia with stem cell characteristics differentiate into retinal ganglion cell (RGC) precursors *in vitro* and partially restore RGC function *in vivo* following transplantation (Singhal et al., 2012)	STEM CELLS TRANSLATIONAL MEDICINE	158	12.2
55	Varadarajan, SG	2022	Central nervous system regeneration (Varadarajan et al., 2022)	CELL	157	52.3
56	Sherpa, T	2008	Ganglion cell regeneration following whole-retina destruction in zebrafish (Sherpa et al., 2008)	DEVELOPMENTAL NEUROBIOLOGY	157	9.2
57	Logan, A	2006	Neurotrophic factor synergy is required for neuronal survival and disinhibited axon regeneration after CNS injury (Logan et al., 2006)	BRAIN	150	7.9
58	Cartoni, R	2016	The mammalian-specific protein armcx1 regulates mitochondrial transport during axon regeneration (Cartoni et al., 2016)	NEURON	150	16.7
59	Jin, ZB	2019	Stemming retinal regeneration with pluripotent stem cells (Jin et al., 2019)	PROGRESS IN RETINAL AND EYE RESEARCH	149	24.8
60	Chierzi, S	2005	The ability of axons to regenerate their growth cones depends on axonal type and age, and is regulated by calcium, cAMP and ERK (Chierzi et al., 2005)	EUROPEAN JOURNAL OF NEUROSCIENCE	149	7.5
61	Pernet, V	2006	Synergistic action of brain-derived neurotrophic factor and lens injury promotes retinal ganglion cell survival, but leads to optic nerve dystrophy *in vivo* (Pernet and Di Polo, 2006)	BRAIN	148	7.8
62	Müller, A	2009	Exogenous CNTF stimulates axon regeneration of retinal ganglion cells partially via endogenous CNTF (Müller et al., 2009)	MOLECULAR AND CELLULAR NEUROSCIENCE	146	9.1
63	Leibinger, M	2013	Interleukin-6 contributes to CNS axon regeneration upon inflammatory stimulation (Leibinger et al., 2013b)	CELL DEATH & DISEASE	146	12.2
64	Berry, M	2008	Regeneration of axons in the visual system (Berry et al., 2008)	RESTORATIVE NEUROLOGY AND NEUROSCIENCE	145	8.5
65	Chung, RS	2008	Redefining the role of metallothionein within the injured brain - Extracellular metallothioneins play an important role in the astrocyte-neuron response to injury (Chung et al., 2008)	JOURNAL OF BIOLOGICAL CHEMISTRY	144	8.5
66	Wu, T	2012	A photon-driven micromotor can direct nerve fiber growth (Wu et al., 2012)	NATURE PHOTONICS	143	11.0
67	Laha, B	2017	Regenerating optic pathways from the eye to the brain (Laha et al., 2017)	SCIENCE	142	17.8
68	Li, YQ	2017	Mobile zinc increases rapidly in the retina after optic nerve injury and regulates ganglion cell survival and optic nerve regeneration (Li et al., 2017)	PROCEEDINGS OF THE NATIONAL ACADEMY OF SCIENCES OF THE UNITED STATES OF AMERICA	141	17.6
69	Luo, XT	2013	Three-dimensional evaluation of retinal ganglion cell axon regeneration and pathfinding in whole mouse tissue after injury (Luo et al., 2013)	EXPERIMENTAL NEUROLOGY	140	11.7
70	Gaub, P	2011	The histone acetyltransferase p300 promotes intrinsic axonal regeneration (Gaub et al., 2011)	BRAIN	138	9.9
71	Williams, PR	2020	Axon regeneration in the mammalian optic nerve (Williams et al., 2020)	ANNUAL REVIEW OF VISION SCIENCE, VOL 6, 2020	137	27.4
72	Fligor, CM	2018	Three-dimensional retinal organoids facilitate the investigation of retinal ganglion cell development, organization and neurite outgrowth from human pluripotent stem cells (Fligor et al., 2018)	SCIENTIFIC REPORTS	137	19.6
73	Fischer, D	2012	Promoting optic nerve regeneration (Fischer and Leibinger, 2012)	PROGRESS IN RETINAL AND EYE RESEARCH	136	10.5
74	Hoffman, PN	2010	A conditioning lesion induces changes in gene expression and axonal transport that enhance regeneration by increasing the intrinsic growth state of axons (Hoffman, 2010)	EXPERIMENTAL NEUROLOGY	135	9.0
75	Hu, Y	2005	Lentiviral-mediated transfer of CNTF to Schwann cells within reconstructed peripheral nerve grafts enhances adult retinal ganglion cell survival and axonal regeneration (Hu et al., 2005)	MOLECULAR THERAPY	131	6.6
76	Baldwin, KT	2015	Neuroinflammation triggered by β-glucan/dectin-1 signaling enables CNS axon regeneration (Baldwin et al., 2015)	PROCEEDINGS OF THE NATIONAL ACADEMY OF SCIENCES OF THE UNITED STATES OF AMERICA	131	13.1
77	Venugopalan, P	2016	Transplanted neurons integrate into adult retinas and respond to light (Venugopalan et al., 2016)	NATURE COMMUNICATIONS	131	14.6
78	Krucoff	2016	Enhancing nervous system recovery through neurobiologics, neural interface training, and neurorehabilitation (Krucoff et al., 2016)	FRONTIERS IN NEUROSCIENCE	129	14.3
79	Dun, XP	2017	Role of netrin-1 signaling in nerve regeneration (Dun and Parkinson, 2017)	INTERNATIONAL JOURNAL OF MOLECULAR SCIENCES	128	16.0
80	Biermann, J	2010	Valproic acid-mediated neuroprotection and regeneration in injured retinal ganglion cells (Biermann et al., 2010)	INVESTIGATIVE OPHTHALMOLOGY & VISUAL SCIENCE	128	8.5
81	Yang, C	2020	Rewiring neuronal glycerolipid metabolism determines the extent of axon regeneration (Yang et al., 2020)	NEURON	125	25.0
82	Qin, S	2013	Cross-talk between KLF4 and STAT3 regulates axon regeneration (Qin et al., 2013)	NATURE COMMUNICATIONS	122	10.2
83	Yang, L	2014	The mTORC1 effectors S6K1 and 4E-BP play different roles in CNS axon regeneration (Yang et al., 2014)	NATURE COMMUNICATIONS	122	11.1
84	Pernet, V	2013	Long-distance axonal regeneration induced by CNTF gene transfer is impaired by axonal misguidance in the injured adult optic nerve (Pernet et al., 2013a)	NEUROBIOLOGY OF DISEASE	121	10.1
85	Bray, ER	2019	Thrombospondin-1 mediates axon regeneration in retinal ganglion cells (Bray et al., 2019)	NEURON	121	20.2
86	Wang, XW	2018	Lin28 signaling supports mammalian pns and cns axon regeneration (Wang et al., 2018)	CELL REPORTS	120	17.1
87	Bollaerts, I	2017	Neuroinflammation as fuel for axonal regeneration in the injured vertebrate central nervous system (Bollaerts et al., 2017)	MEDIATORS OF INFLAMMATION	120	15.0
88	Mead, B	2017	Concise review: dental pulp stem cells: a novel cell therapy for retinal and central nervous system repair (Mead et al., 2017)	STEM CELLS	117	14.6
89	Li, S	2016	Promoting axon regeneration in the adult CNS by modulation of the melanopsin/GPCR signaling (Li et al., 2016)	PROCEEDINGS OF THE NATIONAL ACADEMY OF SCIENCES OF THE UNITED STATES OF AMERICA	117	13.0
90	Cui, Q	2006	Actions of neurotrophic factors and their signaling pathways in neuronal survival and axonal regeneration (Cui, 2006)	MOLECULAR NEUROBIOLOGY	116	6.1
91	King, CE	2007	Erythropoietin is both neuroprotective and neuroregenerative following optic nerve transection (King et al., 2007)	EXPERIMENTAL NEUROLOGY	116	6.4
92	Leibinger, M	2013	Neuronal STAT3 activation is essential for CNTF- and inflammatory stimulation-induced CNS axon regeneration (Leibinger et al., 2013a)	CELL DEATH & DISEASE	115	9.6
93	Zwart, I	2009	Umbilical cord blood mesenchymal stromal cells are neuroprotective and promote regeneration in a rat optic tract model (Zwart et al., 2009)	EXPERIMENTAL NEUROLOGY	115	7.2
94	Fischer, D	2008	Crystallins of the β/γ-superfamily mimic the effects of lens injury and promote axon regeneration (Fischer et al., 2008)	MOLECULAR AND CELLULAR NEUROSCIENCE	114	6.7
95	Harvey, AR	2006	Gene therapy and transplantation in CNS repair: The visual system (Harvey et al., 2006)	PROGRESS IN RETINAL AND EYE RESEARCH	113	5.9
96	Pernet, V	2013	Misguidance and modulation of axonal regeneration by Stat3 and Rho/ROCK signaling in the transparent optic nerve (Pernet et al., 2013b)	CELL DEATH & DISEASE	113	9.4
97	Elsaeidi, F	2014	Jak/Stat signaling stimulates zebrafish optic nerve regeneration and overcomes the inhibitory actions of Socs3 and Sfpq (Elsaeidi et al., 2014)	JOURNAL OF NEUROSCIENCE	112	10.2
98	Yan, L	2016	Aligned nanofibers from polypyrrole/graphene as electrodes for regeneration of optic nerve via electrical stimulation (Yan et al., 2016)	ACS APPLIED MATERIALS & INTERFACES	111	12.3
99	Pastrana, E	2006	Genes associated with adult axon regeneration promoted by olfactory ensheathing cells: a new role for matrix metalloproteinase 2 (Pastrana et al., 2006)	JOURNAL OF NEUROSCIENCE	111	5.8
100	Au, NPB	2022	Neuroinflammation, microglia and implications for retinal ganglion cell survival and axon regeneration in traumatic optic neuropathy (Au and Ma, 2022)	FRONTIERS IN IMMUNOLOGY	103	34.3

### 3.2 Analysis of the most productive countries

**FIGURE 3:**
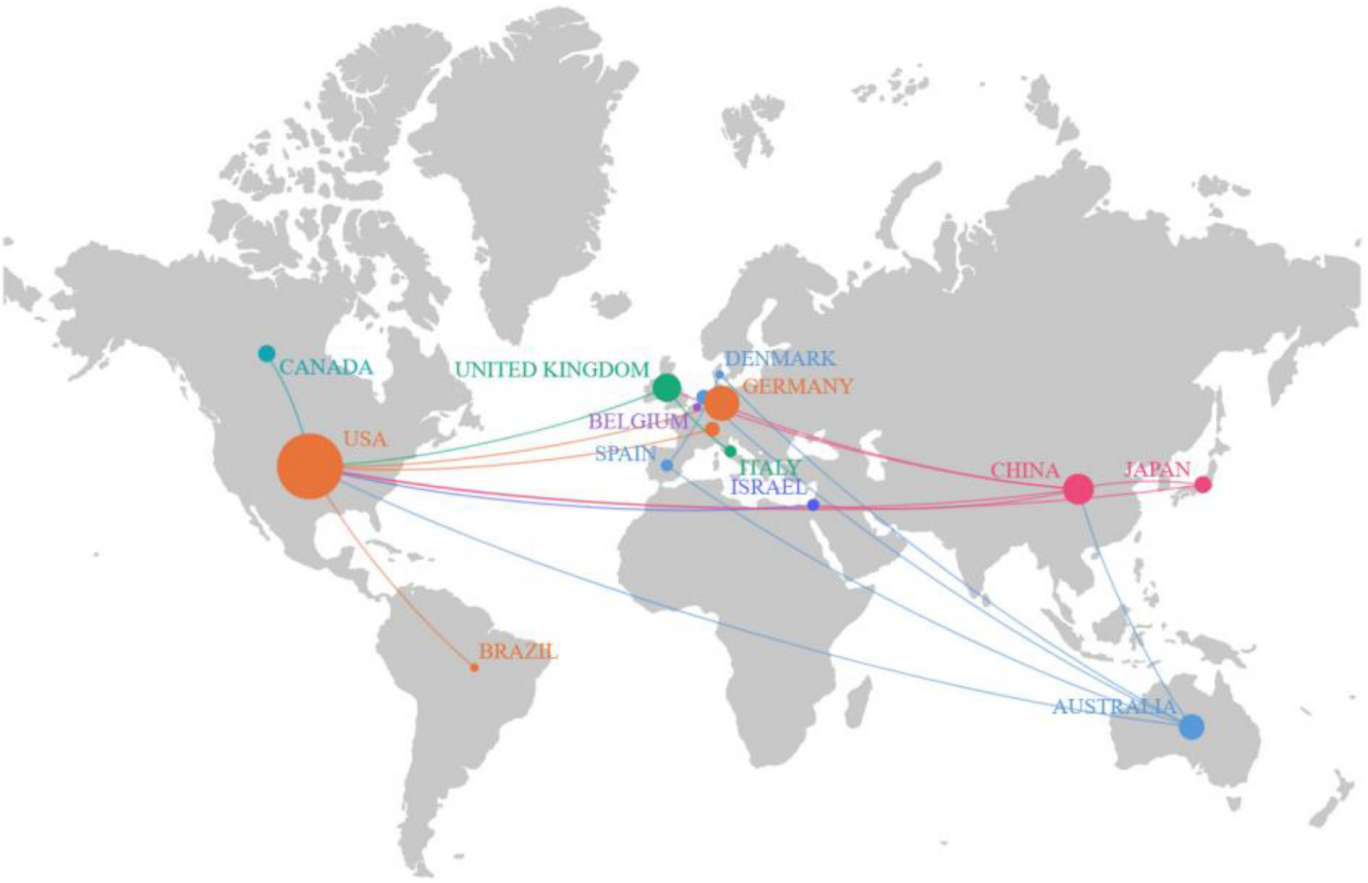
The world map displays a number of national publications.

An analysis of the pattern of international collaboration in optic nerve regeneration research showed that a total of 15 countries/regions participated in the 100 most-cited papers. Figure 3 illustrates the national collaborative network in this field through a world map generated using Scimago Graphica software. The size of the bubbles represents the number of publications in each country, while the thickness of the connecting lines represents the closeness of cooperation between countries. The field of optic nerve regeneration was dominated by the United States, accounting for 59 of the 100 most-cited papers. These publications received a total of 13,981 citations, averaging 237.0 per paper (Table 2). Germany ranked second, contributing 17 papers that received 2,645 citations, resulting in an average of 155.6 citations per paper. China was third, contributing 13 articles. These accumulated a total of 2,076 citations, with an average of 156.0 citations per article. The United States, China, Germany, and the United Kingdom have established significant research collaborations in this area. While other countries also have partnerships, these connections are relatively weak and more fragmented.

**TABLE 2:** The 15 countries in terms of publications and collaborations.

Rank	Country	Publication	Citations	Average citation
1	USA	59	13,981	237.0
2	Germany	17	2,645	155.6
3	China	13	2,076	156.0
4	UK	11	1,389	118.4
5	Australia	9	1,470	163.3
6	Canada	4	836	209.0
7	Japan	4	661	165.3
8	Switzerland	3	471	157.0
9	Israel	3	386	128.7
10	Netherlands	2	424	212.0
11	Spain	2	224	112.0
12	Italy	2	304	152.0
13	Denmark	1	125	125.0
14	Brazil	1	282	282.0
15	Belgium	1	69	69.0

### 3.3 Institution analysis

**FIGURE 4:**
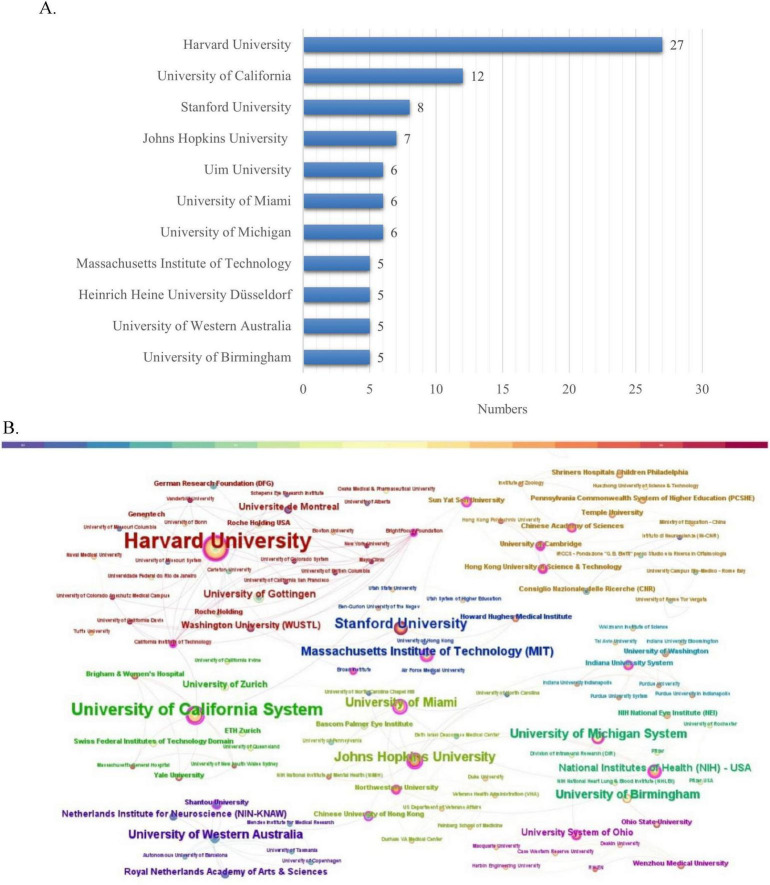
Institution analysis. **(A)** The most relevant institutions. **(B)** Partnerships between institutions.

A total of 134 research institutions worldwide contributed to the 100 most influential papers in the field of optic nerve regeneration. As shown in the institutional ranking in Figure 4A, Harvard University stands out with 30 highly cited papers, establishing it as the most important research force in the field. Cite Space software is used to visualize the interconnections between institutions (Figure 4B). Several prominent research institutions can be identified, such as Harvard University, Boston Children’s Hospital, the University of California, and Stanford University, among others.

### 3.4 Author analysis

**FIGURE 5:**
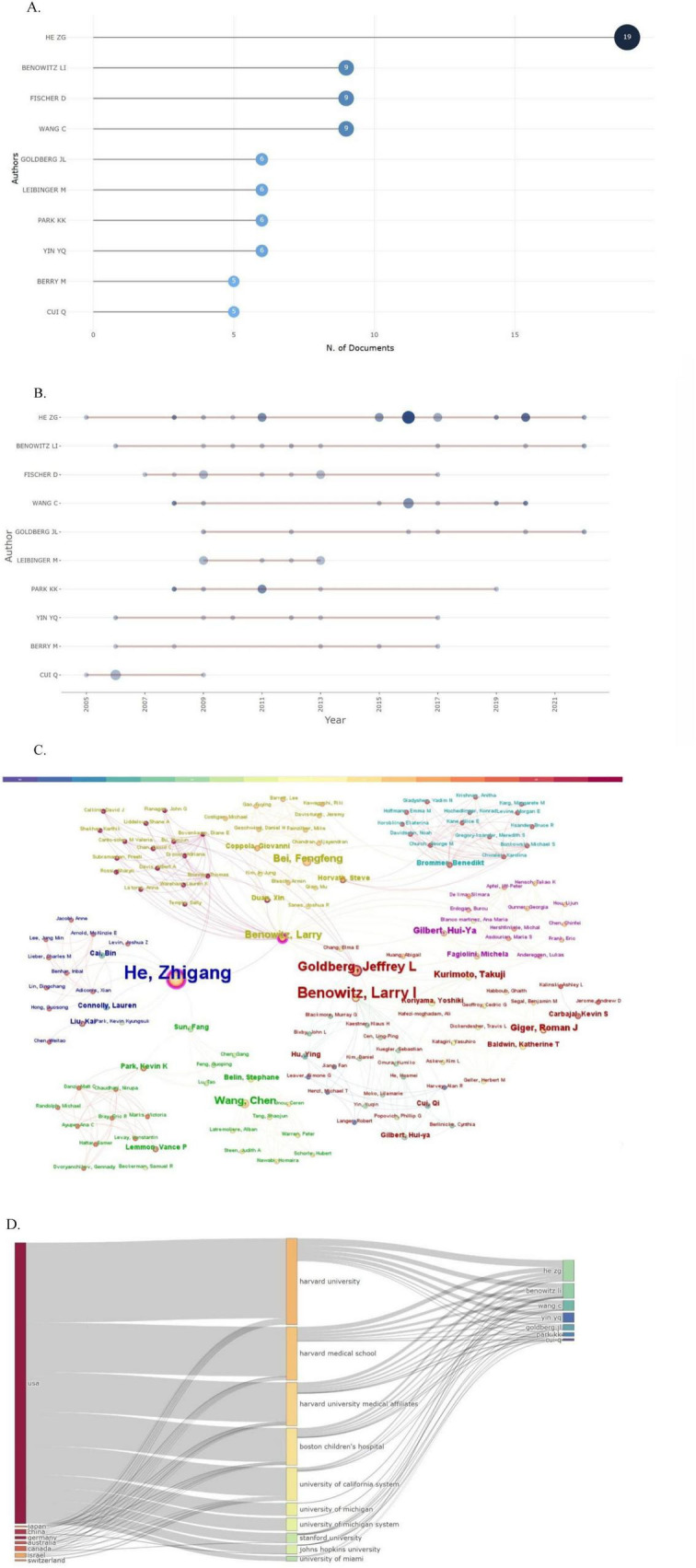
Author analysis. **(A)** Most relevant authors. **(B)** Authors’ production over time. **(C)** The author’s collaborative relationship map. **(D)** Three-field plot (country-affiliation-author).

As shown in Figure 5A, He Zhigang was the most prolific author, contributing to 19 publications. The academic activity period of the top 10 core researchers is presented through a time trend chart in Figure 5B. The cooperative network map in Figure 5C reveals the existence of a continuous and stable network of academic cooperation in this field. The Sankey diagram in Figure 5D further shows the flow of knowledge among countries, institutions, and authors, highlighting the significant academic influence of American researchers in this field.

### 3.5 Journal analysis

**FIGURE 6:**
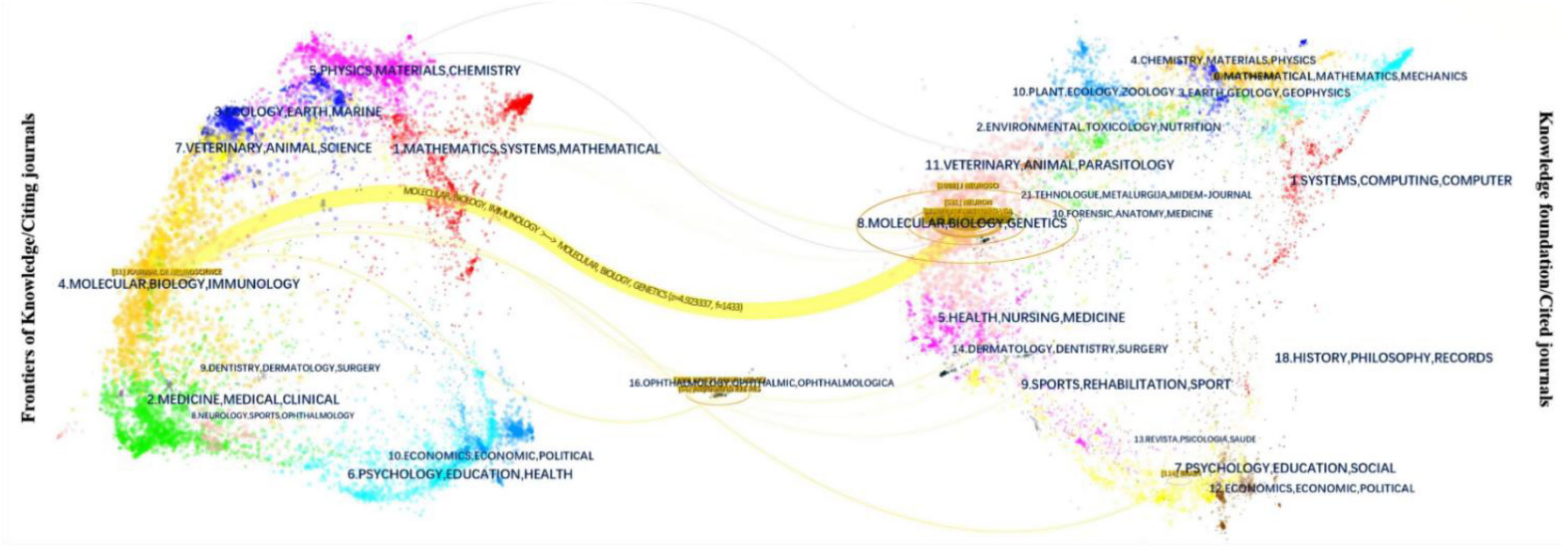
Dual-map overlay of journals.

The 18 journals that have published at least two articles in the field of optic nerve regeneration and their main characteristics are listed in Table 3. The two journals with the most publications in the field of optic nerve regeneration are Journal of Neuroscience and Neuron, with 10 articles, respectively. In addition, four articles were published in Science and two in Cell. The Dual-Map Overlay journal atlas in Figure 6 demonstrates the dynamic knowledge flow from the basic disciplines on the right (represented by cited journals) to the frontier disciplines on the left (represented by citing journals). The frontier research of optic nerve regeneration, primarily concentrated in disciplines on the left such as “MOLECULAR, BIOLOGY, IMMUNOLOGY,” is supported by a solid knowledge base derived from two core disciplinary clusters on the right: one centered on “OPHTHALMOLOGY, OPHTHALMIC, OPHTHALMOLOGICA,” providing the fundamentals of Ophthalmology, and the other centered on “MOLECULAR, BIOLOGY, GENETICS,” forming the immunological basis. The overlay map also shows significant knowledge flow from domains like “CHEMISTRY, MATERIALS, PHYSICS” toward the medical frontiers. This implies that disciplines such as biomaterial, the application of nanotechnology in drug delivery systems, and biophysics provide essential technical tools and innovative solutions for the treatment strategies of optic nerve regeneration.

**TABLE 3:** The journals that have published the 100 most-cited articles in the field of optic nerve regeneration.

Ranking	Journal	Documents	IF in 2023	Total citations	Average citations
1	Neuron	10	14.7	2,479	247.9
2	Journal of neuroscience	10	4.4	1,855	185.5
3	Proceedings of the national academy of sciences of the United States of America	8	9.4	1,855	231.9
4	Brain	5	10.6	782	156.4
5	Experimental neurology	5	4.6	579	115.8
6	Cell death and disease	4	8.1	458	114.5
7	Science	4	44.7	2,291	572.8
8	Progress in retinal and eye research	3	18.6	358	119.3
9	Nature neuroscience	3	21.2	1,000	333.3
10	Current opinion in neurobiology	3	4.8	644	214.7
11	Molecular and cellular neuroscience	3	2.6	392	130.7
12	Nature communications	3	14.7	326	108.7
13	Cell	2	45.5	341	170.5
14	Frontiers in neuroscience	2	3.2	324	162.0
15	Nature	2	50.5	1031	515.5
16	International journal of molecular sciences	2	5.7	260	130.0
17	Investigative ophthalmology and visual science	2	5	275	137.5
18	Stem cells translational medicine	2	5.4	454	227.0

### 3.6 Keywords and research hot spots

**FIGURE 7:**
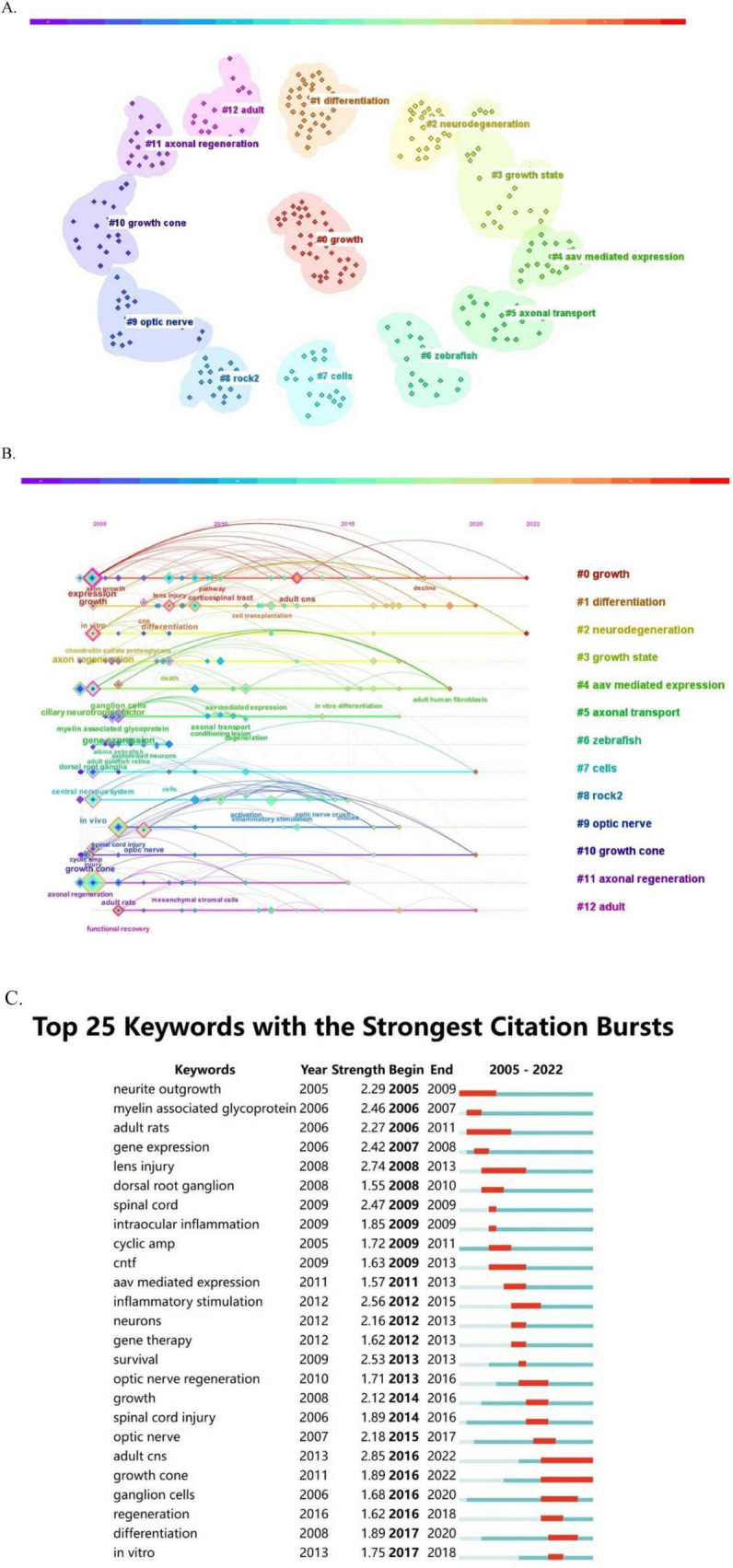
Keyword analysis. **(A)** Keyword cluster graph**. (B)** Timeline view of keywords. **(C)** Burst test of keywords.

Keywords play a crucial role in delineating the focus of an article, giving researchers a clear understanding of the published topic. The co-occurrence of two keywords in a given paper means that there is an intrinsic relationship between them, and the frequency of their occurrence reflects the strength of this connection. Conducting keyword co-occurrence and emergent item analyses allows the identification of the hot topics in different periods in a specific field and the consolidation of the author-provided keywords into a dataset. Keyword clustering describes the inherent knowledge structure within a particular research field and classifies its domain. In this study, cluster analysis revealed that the keywords in the field of optic nerve regeneration can be divided into the following 13 categories (Figure 7A): growth, differentiation, neurodegeneration, growth state, aav mediated expression, axonal transport, zebrafish, cells, rock2, optic nerve, growth cone, axonal regeneration and adult.

The temporal evolution of keyword usage patterns is shown in Figure 7B. The size of each point in Figure 7B is related to the occurrence of the corresponding keyword. The larger the block, the higher the frequency of keyword occurrence. In addition, keywords that exhibit recent growth trends may represent hot research topics in the future. Our keyword burst analysis identified several notable words (Figure 7C), including: “lens injury,” “CNTF,” “myelin-associated glycoprotein,” “neurite outgrowth,” “optic nerve regeneration,” “AAV-mediated expression,” “intraocular inflammation,” “ganglion cells,” and “gene therapy.” Keywords with the strongest burst signal reflect the current research frontier in the field. The keywords of earlier bursts indicate that research interest was initially concentrated in these areas, while more recent bursts denote a marked increase in interest in the topic. Figure 7C highlights the five keywords with the highest burst intensity—“adult CNS,” “lens injury,” “inflammatory stimulation,” “survival,” and “spinal cord”—exhibiting burst intensities of 2.85, 2.74, 2.56, 2.53, and 2.47, respectively. The optic nerve and the spinal cord both belong to the central nervous system and are highly similar in terms of anatomical structure, cellular composition and microenvironment. The two of them share many key technological platforms and material strategies in regenerative medicine. The keywords in the earliest burst were “neurite outgrowth,” “myelin-associated glycoprotein,” “adult rats,” “gene expression,” and “lens injury,” representing the focus of initial research. Adult rats and zebrafish are often used as animal models for optic nerve regeneration and are closely related to the research on the optic nerve. Meanwhile, the keyword that recently showed a burst was “growth cone,” which reflects a new area of intense research interest.

## 4 Discussion

In this study, we conducted a comprehensive data and bibliometric analysis of the 100 most cited publications between 2005 and 2025 in the field of optic nerve regeneration. This analytical strategy enabled a detailed investigation of the evolution, key focus areas, and innovative trends in optic nerve regeneration research, and provided valuable quantitative insights into recent research, thereby deepening the understanding of the topic.

The top 100 publications accumulated a total of 34,636 citations, with citation counts ranging from 103 to 1,545, resulting in a median of 346.4 citations per article. He Zhigang was considered the most productive contributor, having contributed to 19 of these papers. The United States accounted for the highest number of publications (59), followed by Germany and the China with 17 and 13 publications, respectively. The Harvard University system was the most prolific institution, publishing 30 papers, followed by Children’s Hospital and Stanford University, both with eight papers. Keyword analysis identified several areas of interest, including myelin-associated glycoprotein, intraocular inflammation, CNTF, AAV-mediated expression, and gene therapy. Further keyword analysis revealed “growth cone” as an important recent keyword in the field.

### 4.1 Myelin-associated glycoprotein

Myelin-associated glycoprotein (MAG) is a transmembrane protein primarily expressed in myelin-forming cells (oligodendrocytes and Schwann cells) of the CNS and PNS. MAG is an important inhibitor of neurite growth. After optic nerve injury, this glycoprotein accumulates in myelin debris at the injured site, resulting in the formation of a microenvironment unfavorable to axon regeneration (David and Kottis, 1994). MAG has a bidirectional transduction mechanism, namely, myelin-to-axon and axon-to-myelin. In the former, MAG maintains the stability of the myelin–axon interface by specifically binding in *trans* to complex gangliosides, such as GT1b and GD1a, on the surface of the axon membrane. Furthermore, the binding of MAG dimers to sialic acid triggers axon growth cone collapse and inhibits microtubule assembly and disassembly dynamics, thus impeding nerve regeneration (Pronker et al., 2016). The latter (axon-to-myelin) involves the regulation of myelin formation and maintenance, which is dependent on the tyrosine kinase Fyn (Cafferty et al., 2010). Thus, the targeted regulation of MAG is crucial for promoting optic nerve regeneration. Evidence suggests that interventions targeting MAG alone (such as gene knockout or the use of neutralizing antibodies) only weakly promote optic axon regeneration. However, triple knockout of Nogo-A, MAG, and oligodendrocyte-myelin glycoprotein (OMgp) can significantly reduce the collapse of growth cones and extend the regeneration distance of RGC axons after optic nerve injury. (Zhang et al., 2022). Therefore, future studies should prioritize multi-target synergistic treatment strategies combined with novel delivery technologies to overcome the multiple inhibition barriers that impede regeneration in the CNS and ultimately achieve functional optic nerve regeneration.

### 4.2 Ciliary neurotrophic factor

Ciliary neurotrophic factor (CNTF) is mainly secreted by astrocytes during optic nerve regeneration and its endogenous expression can be significantly activated in response to inflammation or nerve injury (Kimura et al., 2016). CNTF is typically delivered via single intravitreal injection. However, its short half-life and the difficulty associated with the maintenance of effective concentrations limit its axonal regeneration effect (Müller et al., 2009). Over recent years, strategies employing subretinal injection mediated by adeno-associated virus (AAV) vectors or the delivery of genetically modified neural stem cells (CNTF-NS) have been developed. These methods have achieved continuous CNTF expression, thereby significantly prolonging the window of opportunity for axon regeneration while avoiding the inflammation induced by repeated injection (Cen et al., 2017; Dulz et al., 2020; Pernet et al., 2013b). Notably, although single CNTF treatment can induce axonal regeneration, functional recovery is limited. Studies have shown that PTEN/SOCS3 gene double-knockout or osteopontin (OPN), insulin-like growth factor 1 (IGF1), and CNTF co-expression can significantly enhance regeneration efficiency through the synergistic activation of downstream signaling pathways (Jacobi et al., 2022). Combining the administration of potassium channel blockers (such as 4-AP) with PTEN/SOCS3 gene knockout can further promote the recovery of axon electrophysiological function (Bei et al., 2016). Recently, Behtaj et al. (2024) innovatively created a bionic delivery system with gradient slow-release properties by covalently coupling CNTF to an electrospun polyglycerol sebacate/polycaprolactone (PGS/PCL) scaffold. The authors reported that this system could guide the directional migration of RGC axons toward regions with high CNTF concentrations, providing a novel therapeutic strategy for optic nerve regeneration (Behtaj et al., 2024).

### 4.3 Intraocular inflammation

Progress in optic nerve regeneration research has highlighted the therapeutic potential of the inflammatory response in regenerative medicine. Classical studies have shown that intraocular inflammatory stimuli, such as lens injury and yeast cell wall (zymosan) injection, can activate the regenerative program in RGCs, thereby circumventing the regenerative limitations inherent to the CNS (Yin et al., 2009). The resulting cytokine cascade triggered by immune cell infiltration is particularly critical in this process. The macrophage-/neutrophil-specific secretion of oncomodulin (OCM) is a central mediator of axonal regeneration. Its levels markedly increase after inflammatory stimulation and functional blockade experiments have shown that OCM specifically regulates axon regeneration without affecting RGC survival (Benowitz and Popovich, 2011; Yin et al., 2009). However, independent validation of these findings with OCM knockout mice has not yet been carried out. This provides a precise entry point for targeted intervention. Importantly, the microenvironment regulatory network has significant synergistic effects. For example, SDF1 enhances OCM activity by upregulating intracellular cAMP levels, thereby forming a multi-factor synergistic mechanism that overcomes the therapeutic bottleneck of single-factor therapy (Xie et al., 2022). The latest breakthrough in the field comes from the establishment of the conditioned lens injury (cLI) model, a non-genetic intervention strategy involving the implementation of mild lens injury two weeks before optic nerve compression (ONC). By recruiting CCR^2+^ immune cell populations, this strategy demonstrated the ability to fully regenerate axons beyond traditional approaches and even achieve functional brain innervation (Feng et al., 2023). Crucially, the regenerative effect of cLI is independent of known factors, such as OCM, suggesting the existence of novel immune regulatory pathways, a finding that may reshape existing theoretical frameworks and open up new research directions. However, the duration and nature of inflammation demonstrate a distinct “double-edged sword” characteristic. Acute inflammatory responses contribute to the regeneration-supportive microenvironment by activating microglia to remove damaged cellular debris and recruiting myeloid cells to secrete neurotrophic factors, such as regulatory proteins and cntf. In contrast, chronic inflammation, through sustained release of neurotoxic mediators, including interleukin-1β (IL-1β) and tumor necrosis factor-α (TNF-α), promotes the activation of inhibitory A1 astrocytes and compromises the integrity of the blood-retinal barrier. These processes disrupt microenvironmental homeostasis, ultimately impeding nerve regeneration and repair (Au and Ma, 2022). This suggests that future studies need to establish an accurate immunophenotypic regulatory system to achieve a dynamic balance between the pro-regeneration mechanism and the neuroprotective effect by controlling the intensity of inflammation. This may promote a paradigm shift in optic nerve repair strategies from empirical intervention to intelligent regulation.

### 4.4 AAV-mediated gene modulation

Adeno-associated virus-mediated gene modulation is a technique that uses AAV as a gene delivery vector to introduce foreign genes into target cells or tissues and enable their expression (Leaver et al., 2006). More than 100 natural AAV serotypes have been identified, of which 13 (including AAV_1–9_ and rh10) have shown an affinity for specific subsets of retinal cells in ophthalmic studies (Carvalho et al., 2018). AAVs can carry genes encoding cytokines (e.g., CNTF, hIL-6) under the regulation of tissue-specific promoters (such as CAG and hSyn), thereby achieving directed gene expression. Studies have shown that the AAV_2_ serotype targets RGCs with high specificity, effectively improving their survival rate and promoting axon regeneration (Cao et al., 2019). However, its transduction range is limited to the local injection and diffusion area (Liu et al., 2020; Ross et al., 2021). Traditional invasive delivery methods, such as intravitreal or subretinal injection, are effective in targeting RGCs but can lead to complications such as retinal detachment and bleeding, as well as result in unequal viral distribution. Recent studies have attempted to target RGCs via the injection of AAV-PHP.eB through the retroorbital venous sinus. This serotype exhibits significantly enhanced transduction efficiency in mouse models. However, AAV-PHP.eB can penetrate the blood–brain barrier and thereby induce off-target effects in the CNS and also has relatively low tissue specificity (Tang et al., 2024). New AAV variants and specific promoters need to be identified to improve targeting accuracy and reduce systemic side effects. Meanwhile, combining these advancements with non-invasive delivery technology and novel cytokines holds promise for overcoming current limitations and achieving multi-gene synergistic therapy.

### 4.5 Growth cone

The growth cone is the core functional structure of axon regeneration, playing a key role in microenvironment perception, signal integration, and guidance extension during nerve injury repair (Chierzi et al., 2005). In models of optic nerve injury, the axonal ends of RGCs in adult mammals often form characteristic retractable ball structures. This pathological phenomenon is considered to be an important morphological sign of hindered axon regeneration. Recent studies have revealed that the knockout of the gene coding for non-muscle myosin IIA/B in RGCs significantly reduced the formation of retractable spheres, and successfully transformed the ends of axons with stagnant regeneration into functional growth cones with dynamic activity, thus achieving a significant improvement in axon regenerative ability (Wang et al., 2020). From a cell biological perspective, axon regeneration requires adequate membrane component support. Studies have shown that enhancing phospholipid synthesis in RGCs by modulating lipid metabolism can effectively promote growth cone membrane extension (Chen et al., 2024). Regarding microenvironment regulation, laminin significantly enhances the structural stability of the growth cone and promotes its continuous extension by activating the integrin receptor signaling pathway (Fligor et al., 2018). In addition, studies on growth cone guiding molecules have demonstrated that netrin-1 and other chemical orientation factors can both significantly improve the axon elongation rate and increase the directional elongation of growth cones by activating intracellular signaling cascades (Qiu et al., 2024).

### 4.6 Clinical translation

The regeneration of the optic nerve is a key part of the restoration of visual function in people with blindness. The convergence of life science and engineering technologies is driving revolutionary breakthroughs in the field of optic nerve regeneration. Multidisciplinary collaborations have led to innovations such as the development of chitosan-CNTF bioactive materials and targeted delivery systems, while gene editing techniques have precisely unlocked the regenerative potential of RGCs. Additionally, stem cell therapy can contribute to restoring the ecological balance in the damaged microenvironment. Animal studies have demonstrated that axons can exceed the regenerative limits of the CNS; however, the central challenge in clinical translation lies in bridging the “precision connectivity gap,” that is, ensuring that regenerated nerve fibers not only grow over long distances but also re-establish functional links with the visual centers of the brain. However, the regenerated axons in optic nerve are almost no myelin sheath around them, and thus cannot conduct action potentials (Bei et al., 2016; Suter et al., 2021). The myelin sheath formation is a crucial step in the recovery of visual function (Del Negro et al., 2023), and it have been demonstrated that increased myelin regeneration of the optic nerve is associated with improved visual function (Henriet et al., 2023). The standardized visual function assessments include Visual Evoked Potentials (VEP), which records electrical activity in the visual cortex to confirm the functional connectivity of the retinocortical pathway; Optomotor Response (OMR), a non-invasive behavioral assay that evaluates gross visual functions like motion perception and contrast sensitivity, essential for validating functional improvements in the cLI model; Pupillary Light Reflex (PLR), which assesses the integrity of the retinocollicular pathway and is suitable for models of proximal optic nerve injury such as traumatic optic neuropathy; and Visual Water Maze (VWM), which measures higher-order visual functions. In addition, AI navigation and intelligent biological scaffolds hold promise for resolving the challenge of the accurate docking of optic nerves, potentially leading to brain–computer interfaces or a hybrid pathway of “bio-digital vision.” The future for patients with optic nerve injury is bright and increasingly promising.

## 5 Limitations

This study had several limitations. Although the Web of Science is the most commonly used database for conducting a literature search, it does not contain all publications. Additionally, relying on citation frequency as a selection criterion can lead to the exclusion of recently published works that may be influential but have not yet accumulated a large number of citations. Moreover, there is a possibility of citation bias because papers from certain institutions or well-known authors may receive more citations than equally valuable works from less prominent sources.

## 6 Conclusion

This study systematically revealed the evolution of the research paradigm and the characteristics of the knowledge graph in the field of optic nerve regeneration through a quantitative analysis of the 100 most highly cited publications from the past 20 years. Bibliometric data showed that United States research institutions continue to lead the field in terms of publication volume, international collaboration networks, and breakthrough output. Early studies focused on identifying the mechanisms underlying endogenous neuronal signaling pathways (PTEN/mTOR, KLF family), while recent research has concentrated on identifying novel targets for optic nerve regeneration and the development of intelligent biomaterials. The transnational cooperation network formed by the United States, China, Germany, Britain, and other countries has significantly accelerated the process of knowledge transformation. The core challenge of current research is how to achieve long-distance regeneration of the optic nerve and reconnect the regenerated axons to the relevant brain regions. Future efforts should be concentrated on developing multi-target therapies against inhibitor molecules and establishing real-time immune monitoring platforms for precise control of inflammation response to improve neural regeneration. These advances will help bridge the gap between basic research and clinical treatment for optic nerve repair.

## Data Availability

The original contributions presented in this study are included in this article/supplementary material, further inquiries can be directed to the corresponding author.

## References

[B1] Abe N. Cavalli V. (2008). Nerve injury signaling. *Curr. Opin. Neurobiol.* 18 276–283. 10.1016/j.conb.2008.06.005 18655834 PMC2633416

[B2] Au N. Ma C. (2022). Neuroinflammation, microglia and implications for retinal ganglion cell survival and axon regeneration in traumatic optic neuropathy. *Front. Immunol.* 13:860070. 10.3389/fimmu.2022.860070 35309305 PMC8931466

[B3] Baldwin K. Carbajal K. Segal B. Giger R. (2015). Neuroinflammation triggered by β-glucan/dectin-1 signaling enables CNS axon regeneration. *Proc. Natl. Acad. Sci. U S A.* 112 2581–2586. 10.1073/pnas.1423221112 25675510 PMC4345569

[B4] Batagelj V. Mrvar A. (2002). ““Pajek - Analysis and visualization of large networks. GRAPH Drawing 2265”,” in *Lecture Notes In Computer Science*, Vol. 2265 eds Mutzel P. Junger M. Leipert S. (Berlin: Springer-Verlag Berlin), 477–478.

[B5] Behtaj S. Karamali F. Najafian S. Masaeli E. Rybachuk M. (2024). Ciliary neurotrophic factor mediated growth of retinal ganglion cell axons on PGS/PCL scaffolds. *Biomed. Mater.* 19 10.1088/1748-605X/ad1bae 38181445

[B6] Bei F. Lee H. Liu X. Gunner G. Jin H. Ma L. (2016). Restoration of visual function by enhancing conduction in regenerated axons. *Cell* 164 219–232. 10.1016/j.cell.2015.11.036 26771493 PMC4863988

[B7] Belin S. Nawabi H. Wang C. Tang S. Latremoliere A. Warren P. (2015). Injury-induced decline of intrinsic regenerative ability revealed by quantitative proteomics. *Neuron* 86 1000–1014. 10.1016/j.neuron.2015.03.060 25937169 PMC4551425

[B8] Benowitz L. Popovich P. (2011). Inflammation and axon regeneration. *Curr. Opin. Neurol.* 24 577–583. 10.1097/WCO.0b013e32834c208d 21968547

[B9] Benowitz L. He Z. Goldberg J. (2017). Reaching the brain: Advances in optic nerve regeneration. *Exp. Neurol.* 287 365–373. 10.1016/j.expneurol.2015.12.015 26746987

[B10] Berry M. Ahmed Z. Lorber B. Douglas M. Logan A. (2008). Regeneration of axons in the visual system. *Restor. Neurol. Neurosci.* 26 147–174. 18820408

[B11] Bertrand J. Winton M. Rodriguez-Hernandez N. Campenot R. McKerracher L. (2005). Application of Rho antagonist to neuronal cell bodies promotes neurite growth in compartmented cultures and regeneration of retinal ganglion cell axons in the optic nerve of adult rats. *J. Neurosci.* 25 1113–1121. 10.1523/JNEUROSCI.3931-04.2005 15689547 PMC6725958

[B12] Biermann J. Grieshaber P. Goebel U. Martin G. Thanos S. Di Giovanni S. (2010). Valproic acid-mediated neuroprotection and regeneration in injured retinal ganglion cells. *Invest. Ophthalmol. Vis. Sci.* 51 526–534. 10.1167/iovs.09-3903 19628741

[B13] Bollaerts I. Van Houcke J. Andries L. De Groef L. Moons L. (2017). Neuroinflammation as fuel for axonal regeneration in the injured vertebrate central nervous system. *Mediators Inflamm.* 2017:9478542. 10.1155/2017/9478542 28203046 PMC5288536

[B14] Bray E. Yungher B. Levay K. Ribeiro M. Dvoryanchikov G. Ayupe A. (2019). Thrombospondin-1 mediates axon regeneration in retinal ganglion cells. *Neuron* 103 642–657.e7. 10.1016/j.neuron.2019.05.044 31255486 PMC6706310

[B15] Cafferty W. Duffy P. Huebner E. Strittmatter S. M. (2010). MAG and OMgp synergize with Nogo-A to restrict axonal growth and neurological recovery after spinal cord trauma. *J. Neurosci.* 30 6825–6837. 10.1523/JNEUROSCI.6239-09.2010 20484625 PMC2883258

[B16] Cao X. Yung J. Mak H. Leung C. (2019). Factors governing the transduction efficiency of adeno-associated virus in the retinal ganglion cells following intravitreal injection. *Gene Ther.* 26 109–120. 10.1038/s41434-019-0060-0 30692605

[B17] Cartoni R. Norsworthy M. Bei F. Wang C. Li S. Zhang Y. (2016). The mammalian-specific protein armcx1 regulates mitochondrial transport during axon regeneration. *Neuron* 92 1294–1307. 10.1016/j.neuron.2016.10.060 28009275 PMC5189716

[B18] Carvalho L. Xiao R. Wassmer S. Langsdorf A. Zinn E. Pacouret S. (2018). Synthetic adeno-associated viral vector efficiently targets mouse and nonhuman primate retina in vivo. *Hum. Gene Ther.* 29 771–784. 10.1089/hum.2017.154 29325457 PMC6066192

[B19] Cen L. Liang J. Chen J. Harvey A. Ng T. Zhang M. (2017). AAV-mediated transfer of RhoA shRNA and CNTF promotes retinal ganglion cell survival and axon regeneration. *Neuroscience* 343 472–482. 10.1016/j.neuroscience.2016.12.027 28017835

[B20] Chandran V. Coppola G. Nawabi H. Omura T. Versano R. Huebner E. (2016). A systems-level analysis of the peripheral nerve intrinsic axonal growth program. *Neuron* 89 956–970. 10.1016/j.neuron.2016.01.034 26898779 PMC4790095

[B21] Chang E. Goldberg J. (2012). Glaucoma 2.0: Neuroprotection, neuroregeneration, neuroenhancement. *Ophthalmology* 119 979–986. 10.1016/j.ophtha.2011.11.003 22349567 PMC3343191

[B22] Chen B. Zhang H. Zhai Q. Li H. Wang C. Wang Y. (2022). Traumatic optic neuropathy: A review of current studies. *Neurosurg. Rev.* 45 1895–1913. 10.1007/s10143-021-01717-9 35034261

[B23] Chen C. (2004). Searching for intellectual turning points: Progressive knowledge domain visualization. *Proc. Natl. Acad. Sci. U S A.* 101 5303–5310. 10.1073/pnas.0307513100 14724295 PMC387312

[B24] Chen W. Wu J. Yang C. Li S. Liu Z. An Y. (2024). Lipin1 depletion coordinates neuronal signaling pathways to promote motor and sensory axon regeneration after spinal cord injury. *Proc. Natl. Acad. Sci. U S A.* 121:e2404395121. 10.1073/pnas.2404395121 39292743 PMC11441493

[B25] Chidlow G. Ebneter A. Wood J. Casson R. (2011). The optic nerve head is the site of axonal transport disruption, axonal cytoskeleton damage and putative axonal regeneration failure in a rat model of glaucoma. *Acta Neuropathol.* 121 737–751. 10.1007/s00401-011-0807-1 21311901 PMC3098991

[B26] Chierzi S. Ratto G. Verma P. Fawcett J. (2005). The ability of axons to regenerate their growth cones depends on axonal type and age, and is regulated by calcium, cAMP and ERK. *Eur. J. Neurosci.* 21 2051–2062. 10.1111/j.1460-9568.2005.04066.x 15869501

[B27] Chung R. Penkowa M. Dittmann J. King C. Bartlett C. Asmussen J. (2008). Redefining the role of metallothionein within the injured brain: Extracellular metallothioneins play an important role in the astrocyte-neuron response to injury. *J. Biol. Chem.* 283 15349–15358. 10.1074/jbc.M708446200 18334482 PMC3258880

[B28] Cui Q. (2006). Actions of neurotrophic factors and their signaling pathways in neuronal survival and axonal regeneration. *Mol. Neurobiol.* 33 155–179. 10.1385/MN:33:2:155 16603794

[B29] David S. Kottis V. Identification of myelin-associated glycoprotein as a major myelin-berived of neurite growth. *Neuron* 13 805–811. 10.1016/0896-6273(94)90247-X 7524558

[B30] de Lima S. Koriyama Y. Kurimoto T. Oliveira J. Yin Y. Li Y. (2012). Full-length axon regeneration in the adult mouse optic nerve and partial recovery of simple visual behaviors. *Proc. Natl. Acad. Sci. U S A.* 109 9149–9154. 10.1073/pnas.1119449109 22615390 PMC3384191

[B31] Del Negro I. Pauletto G. Verriello L. Spadea L. Salati C. Ius T. (2023). Uncovering the genetics and physiology behind optic neuritis. *Genes* 14:2192. 10.3390/genes14122192 38137014 PMC10742654

[B32] Dickendesher T. Baldwin K. Mironova Y. Koriyama Y. Raiker S. Askew K. (2012). NgR1 and NgR3 are receptors for chondroitin sulfate proteoglycans. *Nat. Neurosci.* 15 703–712. 10.1038/nn.3070 22406547 PMC3337880

[B33] Duan X. Qiao M. Bei F. Kim I. He Z. Sanes J. (2015). Subtype-specific regeneration of retinal ganglion cells following axotomy: Effects of osteopontin and mTOR signaling. *Neuron* 85 1244–1256. 10.1016/j.neuron.2015.02.017 25754821 PMC4391013

[B34] Dulz S. Bassal M. Flachsbarth K. Riecken K. Fehse B. Schlichting S. (2020). Intravitreal co-administration of GDNF and CNTF confers synergistic and long-lasting protection against injury-induced cell death of retinal ganglion *Cells* in mice. *Cells* 9:2082. 10.3390/cells9092082 32932933 PMC7565883

[B35] Dun X. Parkinson D. (2017). Role of Netrin-1 signaling in nerve regeneration. *Int. J. Mol. Sci.* 18:491. 10.3390/ijms18030491 28245592 PMC5372507

[B36] Ellis-Behnke R. Liang Y. You S. Tay D. Zhang S. So K. (2006). Nano neuro knitting: Peptide nanofiber scaffold for brain repair and axon regeneration with functional return of vision. *Proc. Natl. Acad. Sci. U S A.* 103 5054–5059. 10.1073/pnas.0600559103 16549776 PMC1405623

[B37] Elsaeidi F. Bemben M. Zhao X. Goldman D. (2014). Jak/Stat signaling stimulates zebrafish optic nerve regeneration and overcomes the inhibitory actions of Socs3 and Sfpq. *J. Neurosci.* 34 2632–2644. 10.1523/JNEUROSCI.3898-13.2014 24523552 PMC3921430

[B38] Fausett B. Goldman D. A. (2006). role for alpha1 tubulin-expressing Müller glia in regeneration of the injured zebrafish retina. *J. Neurosci.* 26 6303–6313. 10.1523/JNEUROSCI.0332-06.2006 16763038 PMC6675181

[B39] Feng Q. Wong K. Benowitz L. (2023). Full-length optic nerve regeneration in the absence of genetic manipulations. *JCI Insight* 8:e164579. 10.1172/jci.insight.164579 36821399 PMC10132151

[B40] Fimbel S. Montgomery J. Burket C. Hyde D. (2007). Regeneration of inner retinal neurons after intravitreal injection of ouabain in zebrafish. *J. Neurosci.* 27 1712–1724. 10.1523/JNEUROSCI.5317-06.2007 17301179 PMC6673754

[B41] Fischer D. Leibinger M. (2012). Promoting optic nerve regeneration. *Prog. Retin. Eye Res.* 31 688–701. 10.1016/j.preteyeres.2012.06.005 22781340

[B42] Fischer D. Hauk T. Müller A. Thanos S. (2008). Crystallins of the beta/gamma-superfamily mimic the effects of lens injury and promote axon regeneration. *Mol. Cell. Neurosci.* 37 471–479. 10.1016/j.mcn.2007.11.002 18178099

[B43] Fligor C. Langer K. Sridhar A. Ren Y. Shields P. Edler M. (2018). Three-dimensional retinal organoids facilitate the investigation of retinal ganglion cell development, organization and neurite outgrowth from human pluripotent stem cells. *Sci. Rep.* 8:14520. 10.1038/s41598-018-32871-8 30266927 PMC6162218

[B44] Fujita Y. Yamashita T. (2014). Axon growth inhibition by RhoA/ROCK in the central nervous system. *Front. Neurosci.* 8:338. 10.3389/fnins.2014.00338 25374504 PMC4205828

[B45] Gaub P. Joshi Y. Wuttke A. Naumann U. Schnichels S. Heiduschka P. (2011). The histone acetyltransferase p300 promotes intrinsic axonal regeneration. *Brain* 134 2134–2148. 10.1093/brain/awr142 21705428

[B46] Harvey A. Hu Y. Leaver S. Mellough C. Park K. Verhaagen J. (2006). Gene therapy and transplantation in CNS repair: The visual system. *Prog. Retin. Eye Res*. 25 449–489. 10.1016/j.preteyeres.2006.07.002 16963308

[B47] Hassan-Montero Y. De-Moya-Anegon F. Guerrero-Bote V. P. (2022). *SCImago Graphica*: A new too for exploring and visually communicating data. *Prof. Inf.* 31:e310502. 10.3145/epi.2022.sep.02

[B48] He Z. Jin Y. (2016). Intrinsic control of axon regeneration. *Neuron* 90 437–451. 10.1016/j.neuron.2016.04.022 27151637

[B49] Henriet E. Martin E. Jubin P. Langui D. Mannioui A. Stankoff B. (2023). Monitoring recovery after CNS demyelination, a novel tool to de-risk pro-remyelinating strategies. *Brain* 146 2453–2463. 10.1093/brain/awad051 36995973 PMC10232271

[B50] Hilla A. Diekmann H. Fischer D. (2017). Microglia are irrelevant for neuronal degeneration and axon regeneration after acute injury. *J. Neurosci.* 37 6113–6124. 10.1523/JNEUROSCI.0584-17.2017 28539419 PMC6596505

[B51] Hoffman P. N. A. (2010). conditioning lesion induces changes in gene expression and axonal transport that enhance regeneration by increasing the intrinsic growth state of axons. *Exp. Neurol.* 223 11–18. 10.1016/j.expneurol.2009.09.006 19766119

[B52] Hu Y. Leaver S. Plant G. Hendriks W. Niclou S. Verhaagen J. (2005). Lentiviral-mediated transfer of CNTF to schwann cells within reconstructed peripheral nerve grafts enhances adult retinal ganglion cell survival and axonal regeneration. *Mol. Ther.* 11 906–915. 10.1016/j.ymthe.2005.01.016 15922961

[B53] Jacobi A. Tran N. Yan W. Benhar I. Tian F. Schaffer R. (2022). Overlapping transcriptional programs promote survival and axonal regeneration of injured retinal ganglion cells. *Neuron* 110 2625–2645.e7. 10.1016/j.neuron.2022.06.002 35767994 PMC9391321

[B54] Jin Z. Gao M. Deng W. Wu K. Sugita S. Mandai M. (2019). Stemming retinal regeneration with pluripotent stem cells. *Prog. Retin Eye Res.* 69 38–56. 10.1016/j.preteyeres.2018.11.003 30419340

[B55] Kimura A. Namekata K. Guo X. Harada C. Harada T. (2016). Neuroprotection, growth factors and BDNF-TrkB signalling in retinal degeneration. *Int. J. Mol. Sci.* 17:1584. 10.3390/ijms17091584 27657046 PMC5037849

[B56] King C. Rodger J. Bartlett C. Esmaili T. Dunlop S. Beazley L. (2007). Erythropoietin is both neuroprotective and neuroregenerative following optic nerve transection. *Exp. Neurol.* 205 48–55. 10.1016/j.expneurol.2007.01.017 17328893

[B57] Koch J. Tönges L. Barski E. Michel U. Bähr M. Lingor P. (2014). ROCK2 is a major regulator of axonal degeneration, neuronal death and axonal regeneration in the CNS. *Cell. Death Dis.* 5:e1225. 10.1038/cddis.2014.191 24832597 PMC4047920

[B58] Koprivica V. Cho K. Park J. Yiu G. Atwal J. Gore B. (2005). EGFR activation mediates inhibition of axon regeneration by myelin and chondroitin sulfate proteoglycans. *Science* 310 106–110. 10.1126/science.1115462 16210539

[B59] Kretz A. Happold C. Marticke J. Isenmann S. (2005). Erythropoietin promotes regeneration of adult CNS neurons via Jak2/Stat3 and PI3K/AKT pathway activation. *Mol. Cell. Neurosci.* 29 569–579. 10.1016/j.mcn.2005.04.009 15936213

[B60] Krucoff M. Rahimpour S. Slutzky M. Edgerton V. Turner D. (2016). Enhancing nervous system recovery through neurobiologics, neural interface training, and neurorehabilitation. *Front. Neurosci.* 10:584. 10.3389/fnins.2016.00584 28082858 PMC5186786

[B61] Kurimoto T. Yin Y. Habboub G. Gilbert H. Li Y. Nakao S. (2013). Neutrophils express oncomodulin and promote optic nerve regeneration. *J. Neurosci.* 33 14816–14824. 10.1523/JNEUROSCI.5511-12.2013 24027282 PMC3771038

[B62] Kurimoto T. Yin Y. Omura K. Gilbert H. Kim D. Cen L. (2010). Long-distance axon regeneration in the mature optic nerve: Contributions of oncomodulin, cAMP, and pten gene deletion. *J. Neurosci.* 30 15654–15663. 10.1523/JNEUROSCI.4340-10.2010 21084621 PMC3001271

[B63] Laha B. Stafford B. Huberman A. (2017). Regenerating optic pathways from the eye to the brain. *Science* 356 1031–1034. 10.1126/science.aal5060 28596336 PMC6333302

[B64] Lamba D. Karl M. Reh T. (2008). Neural regeneration and cell replacement: A view from the eye. *Cell. Stem. Cell.* 2 538–549. 10.1016/j.stem.2008.05.002 18522847 PMC2692223

[B65] Lambiase A. Aloe L. Centofanti M. Parisi V. Báo S. Mantelli F. (2009). Experimental and clinical evidence of neuroprotection by nerve growth factor eye drops: Implications for glaucoma. *Proc. Natl. Acad. Sci. U S A.* 106 13469–13474. 10.1073/pnas.0906678106 19805021 PMC2726400

[B66] Leaver S. Cui Q. Plant G. Arulpragasam A. Hisheh S. Verhaagen J. (2006). AAV-mediated expression of CNTF promotes long-term survival and regeneration of adult rat retinal ganglion cells. *Gene Ther.* 13 1328–1341. 10.1038/sj.gt.3302791 16708079

[B67] Leibinger M. Andreadaki A. Diekmann H. Fischer D. (2013a). Neuronal STAT3 activation is essential for CNTF- and inflammatory stimulation-induced CNS axon regeneration. *Cell. Death Dis.* 4:e805. 10.1038/cddis.2013.310 24052073 PMC3789169

[B68] Leibinger M. Müller A. Andreadaki A. Hauk T. Kirsch M. Fischer D. (2009). Neuroprotective and axon growth-promoting effects following inflammatory stimulation on mature retinal ganglion cells in mice depend on ciliary neurotrophic factor and leukemia inhibitory factor. *J. Neurosci.* 29 14334–14341. 10.1523/JNEUROSCI.2770-09.2009 19906980 PMC6665071

[B69] Leibinger M. Müller A. Gobrecht P. Diekmann H. Andreadaki A. Fischer D. (2013b). Interleukin-6 contributes to CNS axon regeneration upon inflammatory stimulation. *Cell. Death Dis.* 4:e609. 10.1038/cddis.2013.126 23618907 PMC3641349

[B70] Li S. Yang C. Zhang L. Gao X. Wang X. Liu W. (2016). Promoting axon regeneration in the adult CNS by modulation of the melanopsin/GPCR signaling. *Proc. Natl. Acad. Sci. U S A.* 113 1937–1942. 10.1073/pnas.1523645113 26831088 PMC4763730

[B71] Li Y. Andereggen L. Yuki K. Omura K. Yin Y. Gilbert H. (2017). Mobile zinc increases rapidly in the retina after optic nerve injury and regulates ganglion cell survival and optic nerve regeneration. *Proc. Natl. Acad. Sci. U S A.* 114 E209–E218. 10.1073/pnas.1616811114 28049831 PMC5240690

[B72] Lim J. Stafford B. Nguyen P. Lien B. Wang C. Zukor K. (2016). Neural activity promotes long-distance, target-specific regeneration of adult retinal axons. *Nat. Neurosci.* 19 1073–1084. 10.1038/nn.4340 27399843 PMC5708130

[B73] Lingor P. Teusch N. Schwarz K. Mueller R. Mack H. Bähr M. (2007). Inhibition of Rho kinase (ROCK) increases neurite outgrowth on chondroitin sulphate proteoglycan in vitro and axonal regeneration in the adult optic nerve in vivo. *J. Neurochem.* 103 181–189. 10.1111/j.1471-4159.2007.04756.x 17608642

[B74] Lingor P. Tönges L. Pieper N. Bermel C. Barski E. Planchamp V. (2008). ROCK inhibition and CNTF interact on intrinsic signalling pathways and differentially regulate survival and regeneration in retinal ganglion cells. *Brain* 131 250–263. 10.1093/brain/awm284 18063589

[B75] Liu K. Tedeschi A. Park K. He Z. (2011). Neuronal intrinsic mechanisms of axon regeneration. *Annu. Rev. Neurosci.* 34 131–152. 10.1146/annurev-neuro-061010-113723 21438684

[B76] Liu Y. Huang S. Ng T. Liang J. Xu Y. Chen S. (2020). Longitudinal evaluation of immediate inflammatory responses after intravitreal AAV2 injection in rats by optical coherence tomography. *Exp. Eye Res.* 193:107955. 10.1016/j.exer.2020.107955 32017940

[B77] Logan A. Ahmed Z. Baird A. Gonzalez A. Berry M. (2006). Neurotrophic factor synergy is required for neuronal survival and disinhibited axon regeneration after CNS injury. *Brain* 129 490–502. 10.1093/brain/awh706 16339795

[B78] Lu Y. Brommer B. Tian X. Krishnan A. Meer M. Wang C. (2020). Reprogramming to recover youthful epigenetic information and restore vision. *Nature* 588 124–129. 10.1038/s41586-020-2975-4 33268865 PMC7752134

[B79] Luo X. Salgueiro Y. Beckerman S. Lemmon V. Tsoulfas P. Park K. (2013). Three-dimensional evaluation of retinal ganglion cell axon regeneration and pathfinding in whole mouse tissue after injury. *Exp. Neurol.* 247 653–662. 10.1016/j.expneurol.2013.03.001 23510761 PMC3726550

[B80] Mead B. Tomarev S. (2017). Bone marrow-derived mesenchymal stem cells-derived exosomes promote survival of retinal ganglion cells through miRNA-dependent mechanisms. *Stem Cells Transl. Med.* 6 1273–1285. 10.1002/sctm.16-0428 28198592 PMC5442835

[B81] Mead B. Berry M. Logan A. Scott R. Leadbeater W. Scheven B. (2015). Stem cell treatment of degenerative eye disease. *Stem Cell. Res.* 14 243–257. 10.1016/j.scr.2015.02.003 25752437 PMC4434205

[B82] Mead B. Logan A. Berry M. Leadbeater W. Scheven B. (2017). Concise review: Dental pulp *Stem Cells*: A novel cell therapy for retinal and central nervous system repair. *Stem Cells* 35 61–67. 10.1002/stem.2398 27273755

[B83] Mead B. Logan A. Berry M. Leadbeater W. Scheven B. (2013). Intravitreally transplanted dental pulp stem cells promote neuroprotection and axon regeneration of retinal ganglion cells after optic nerve injury. *Invest. Ophthalmol. Vis. Sci.* 54 7544–7556. 10.1167/iovs.13-13045 24150755

[B84] Moore D. Blackmore M. Hu Y. Kaestner K. Bixby J. Lemmon V. (2009). KLF family members regulate intrinsic axon regeneration ability. *Science* 326 298–301. 10.1126/science.1175737 19815778 PMC2882032

[B85] Müller A. Hauk T. Fischer D. (2007). Astrocyte-derived CNTF switches mature RGCs to a regenerative state following inflammatory stimulation. *Brain* 130 3308–3320. 10.1093/brain/awm257 17971355

[B86] Müller A. Hauk T. Leibinger M. Marienfeld R. Fischer D. (2009). Exogenous CNTF stimulates axon regeneration of retinal ganglion cells partially via endogenous CNTF. *Mol. Cell. Neurosci.* 41 233–246. 10.1016/j.mcn.2009.03.002 19332123

[B87] Norsworthy M. Bei F. Kawaguchi R. Wang Q. Tran N. Li Y. (2017). Sox11 expression promotes regeneration of some retinal ganglion cell types but kills others. *Neuron* 94 1112–1120.e4. 10.1016/j.neuron.2017.05.035 28641110 PMC5519288

[B88] Pan T. Huang Y. Wei J. Lai C. Chen Y. Nan K. (2024). Implantation of biomimetic polydopamine nanocomposite scaffold promotes optic nerve regeneration through modulating inhibitory microenvironment. *J. Nanobiotechnol.* 22:683. 10.1186/s12951-024-02962-y 39506841 PMC11542345

[B89] Park K. Liu K. Hu Y. Smith P. Wang C. Cai B. (2008). Promoting axon regeneration in the adult CNS by modulation of the PTEN/mTOR pathway. *Science* 322 963–966. 10.1126/science.1161566 18988856 PMC2652400

[B90] Pastrana E. Moreno-Flores M. Gurzov E. Avila J. Wandosell F. Diaz-Nido J. (2006). Genes associated with adult axon regeneration promoted by olfactory ensheathing cells: A new role for matrix metalloproteinase 2. *J. Neurosci.* 26 5347–5359. 10.1523/JNEUROSCI.1111-06.2006 16707787 PMC6675307

[B91] Pernet V. Di Polo A. (2006). Synergistic action of brain-derived neurotrophic factor and lens injury promotes retinal ganglion cell survival, but leads to optic nerve dystrophy in vivo. *Brain* 129 1014–1026. 10.1093/brain/awl015 16418178

[B92] Pernet V. Joly S. Dalkara D. Jordi N. Schwarz O. Christ F. (2013a). Long-distance axonal regeneration induced by CNTF gene transfer is impaired by axonal misguidance in the injured adult optic nerve. *Neurobiol. Dis.* 51 202–213. 10.1016/j.nbd.2012.11.011 23194670

[B93] Pernet V. Joly S. Jordi N. Dalkara D. Guzik-Kornacka A. Flannery J. (2013b). Misguidance and modulation of axonal regeneration by Stat3 and Rho/ROCK signaling in the transparent optic nerve. *Cell. Death Dis.* 4:e734. 10.1038/cddis.2013.266 23868067 PMC3730436

[B94] Pronker M. Lemstra S. Snijder J. Heck A. Thies-Weesie D. Pasterkamp R. (2016). Structural basis of myelin-associated glycoprotein adhesion and signalling. *Nat. Commun.* 7:13584. 10.1038/ncomms13584 27922006 PMC5150538

[B95] Qin S. Zou Y. Zhang C. (2013). Cross-talk between KLF4 and STAT3 regulates axon regeneration. *Nat. Commun.* 4:2633. 10.1038/ncomms3633 24129709 PMC3867821

[B96] Qiu Z. Minegishi T. Aoki D. Abe K. Baba K. Inagaki N. (2024). Adhesion-clutch between DCC and netrin-1 mediates netrin-1-induced axonal haptotaxis. *Front. Mol. Neurosci.* 17:1307755. 10.3389/fnmol.2024.1307755 38375502 PMC10875621

[B97] Ross A. McDougald D. Khan R. Duong T. Dine K. Aravand P. (2021). Rescue of retinal ganglion cells in optic nerve injury using cell-selective AAV mediated delivery of SIRT1. *Gene Ther.* 28 256–264. 10.1038/s41434-021-00219-z 33589779 PMC8149296

[B98] Sas A. Carbajal K. Jerome A. Menon R. Yoon C. Kalinski A. (2020). A new neutrophil subset promotes CNS neuron survival and axon regeneration. *Nat. Immunol.* 21 1496–1505. 10.1038/s41590-020-00813-0 33106668 PMC7677206

[B99] Schwab M. Strittmatter S. (2014). Nogo limits neural plasticity and recovery from injury. *Curr. Opin. Neurobiol.* 27 53–60. 10.1016/j.conb.2014.02.011 24632308 PMC4122629

[B100] Sengottuvel V. Leibinger M. Pfreimer M. Andreadaki A. Fischer D. (2011). Taxol facilitates axon regeneration in the mature CNS. *J. Neurosci.* 31 2688–2699. 10.1523/JNEUROSCI.4885-10.2011 21325537 PMC6623712

[B101] Sherpa T. Fimbel S. Mallory D. Maaswinkel H. Spritzer S. Sand J. (2008). Ganglion cell regeneration following whole-retina destruction in zebrafish. *Dev. Neurobiol.* 68 166–181. 10.1002/dneu.20568 18000816 PMC2581885

[B102] Singhal S. Bhatia B. Jayaram H. Becker S. Jones M. Cottrill P. (2012). Human Müller glia with stem cell characteristics differentiate into retinal ganglion cell (RGC) precursors in vitro and partially restore RGC function in vivo following transplantation. Stem. Cells Transl. Med. 1 188–199. 10.5966/sctm.2011-0005 23197778 PMC3659849

[B103] Smith P. Sun F. Park K. Cai B. Wang C. Kuwako K. (2009). SOCS3 deletion promotes optic nerve regeneration in vivo. *Neuron* 64 617–623. 10.1016/j.neuron.2009.11.021 20005819 PMC2796263

[B104] Sun F. He Z. (2010). Neuronal intrinsic barriers for axon regeneration in the adult CNS. *Curr. Opin. Neurobiol.* 20 510–518. 10.1016/j.conb.2010.03.013 20418094 PMC2911501

[B105] Sun F. Park K. Belin S. Wang D. Lu T. Chen G. (2011). Sustained axon regeneration induced by co-deletion of PTEN and SOCS3. *Nature* 480 372–375. 10.1038/nature10594 22056987 PMC3240702

[B106] Suter T. Wang J. Meng H. He Z. (2021). Utilizing mouse optic nerve crush to examine CNS remyelination. *STAR Protoc.* 2:100796. 10.1016/j.xpro.2021.100796 34786561 PMC8579818

[B107] Tang M. Zhong L. Rong H. Li K. Ye M. Peng J. (2024). Efficient retinal ganglion cells transduction by retro-orbital venous sinus injection of AAV-PHP.eB in mature mice. *Exp. Eye Res.* 244:109931. 10.1016/j.exer.2024.109931 38763353

[B108] Tran N. Shekhar K. Whitney I. Jacobi A. Benhar I. Hong G. (2019). Single-Cell profiles of retinal ganglion cells differing in resilience to injury reveal neuroprotective genes. *Neuron* 104 1039–1055.e12. 10.1016/j.neuron.2019.11.006 31784286 PMC6923571

[B109] van Eck N. Waltman L. (2010). Software survey: Vosviewer, a computer program for bibliometric mapping. *Scientometrics* 84 523–538. 10.1007/s11192-009-0146-3 20585380 PMC2883932

[B110] Varadarajan S. Hunyara J. Hamilton N. Kolodkin A. Huberman A. (2022). Central nervous system regeneration. *Cell* 185 77–94. 10.1016/j.cell.2021.10.029 34995518 PMC10896592

[B111] Veldman M. Bemben M. Thompson R. Goldman D. (2007). Gene expression analysis of zebrafish retinal ganglion cells during optic nerve regeneration identifies KLF6a and KLF7a as important regulators of axon regeneration. *Dev. Biol.* 312 596–612. 10.1016/j.ydbio.2007.09.019 17949705

[B112] Venugopalan P. Wang Y. Nguyen T. Huang A. Muller K. Goldberg J. (2016). Transplanted neurons integrate into adult retinas and respond to light. *Nat. Commun.* 7:10472. 10.1038/ncomms10472 26843334 PMC4742891

[B113] Wang X. Li Q. Liu C. Hall P. Jiang J. Katchis C. (2018). Lin28 signaling supports mammalian PNS and CNS axon regeneration. *Cell. Rep.* 24 2540–2552.e6. 10.1016/j.celrep.2018.07.105 30184489 PMC6173831

[B114] Wang X. Yang S. Zhang C. Hu M. Qian J. Ma J. (2020). Knocking out non-muscle myosin II in retinal ganglion cells promotes long-distance optic nerve regeneration. *Cell. Rep.* 31:107537. 10.1016/j.celrep.2020.107537 32320663 PMC7219759

[B115] Wareham L. Liddelow S. Temple S. Benowitz L. Di Polo A. Wellington C. (2022). Solving neurodegeneration: Common mechanisms and strategies for new treatments. *Mol. Neurodegener.* 17:23. 10.1186/s13024-022-00524-0 35313950 PMC8935795

[B116] Watkins T. Wang B. Huntwork-Rodriguez S. Yang J. Jiang Z. Eastham-Anderson J. (2013). DLK initiates a transcriptional program that couples apoptotic and regenerative responses to axonal injury. *Proc. Natl. Acad. Sci. U S A.* 110 4039–4044. 10.1073/pnas.1211074110 23431164 PMC3593899

[B117] Williams P. Benowitz L. Goldberg J. He Z. (2020). Axon regeneration in the mammalian optic nerve. *Annu. Rev. Vis. Sci.* 6 195–213. 10.1146/annurev-vision-022720-094953 32936739

[B118] Wu T. Nieminen T. Mohanty S. Miotke J. Meyer R. Rubinszein D. (2012). A photon-driven micromotor can direct nerve fibre growth. *Nat. Photonics* 6 62–67. 10.1038/NPHOTON.2011.287

[B119] Xie L. Cen L. Li Y. Gilbert H. Strelko O. Berlinicke C. (2022). Monocyte-derived SDF1 supports optic nerve regeneration and alters retinal ganglion cells’ response to Pten deletion. *Proc. Natl. Acad. Sci. U S A.* 119:e2113751119. 10.1073/pnas.2113751119 35394873 PMC9169637

[B120] Yan L. Zhao B. Liu X. Li X. Zeng C. Shi H. (2016). Aligned nanofibers from polypyrrole/graphene as electrodes for regeneration of optic nerve via electrical stimulation. *ACS Appl. Mater. Interfaces* 8 6834–6840. 10.1021/acsami.5b12843 26926578

[B121] Yang C. Wang X. Wang J. Wang X. Chen W. Lu N. (2020). Rewiring neuronal glycerolipid metabolism determines the extent of axon regeneration. *Neuron* 105 276–292.e5. 10.1016/j.neuron.2019.10.009 31786011 PMC6975164

[B122] Yang L. Miao L. Liang F. Huang H. Teng X. Li S. (2014). The mTORC1 effectors S6K1 and 4E-BP play different roles in CNS axon regeneration. *Nat. Commun.* 5:5416. 10.1038/ncomms6416 25382660 PMC4228696

[B123] Yin Y. Cui Q. Gilbert H. Yang Y. Yang Z. Berlinicke C. (2009). Oncomodulin links inflammation to optic nerve regeneration. *Proc. Natl. Acad. Sci. U S A.* 106 19587–19592. 10.1073/pnas.0907085106 19875691 PMC2780793

[B124] Yin Y. Henzl M. Lorber B. Nakazawa T. Thomas T. Jiang F. (2006). Oncomodulin is a macrophage-derived signal for axon regeneration in retinal ganglion cells. *Nat. Neurosci.* 9 843–852. 10.1038/nn1701 16699509

[B125] Zhang Q. Li Y. Zhuo Y. (2022). Synaptic or non-synaptic? Different intercellular interactions with retinal ganglion cells in optic nerve regeneration. *Mol. Neurobiol.* 59 3052–3072. 10.1007/s12035-022-02781-y 35266115 PMC9016027

[B126] Zhou F. Snider W. (2006). Intracellular control of developmental and regenerative axon growth. *Philos. Trans. R. Soc. Lond. B Biol. Sci.* 361 1575–1592. 10.1098/rstb.2006.1882 16939976 PMC1664665

[B127] Zwart I. Hill A. Al-Allaf F. Shah M. Girdlestone J. Sanusi A. (2009). Umbilical cord blood mesenchymal stromal cells are neuroprotective and promote regeneration in a rat optic tract model. *Exp. Neurol.* 216 439–448. 10.1016/j.expneurol.2008.12.028 19320003

